# Cancer theragnostics: closing the loop for advanced personalized cancer treatment through the platform integration of therapeutics and diagnostics

**DOI:** 10.3389/fbioe.2024.1499474

**Published:** 2025-01-17

**Authors:** Olga V. Sergeeva, Liang Luo, Anthony Guiseppi-Elie

**Affiliations:** ^1^ Genterra JSC, Moscow, Russia; ^2^ National Engineering Research Center for Nanomedicine, College of Life Science and Technology, Huazhong University of Science and Technology, Wuhan, China; ^3^ Bioelectronics, Biosensors and Biochips (C3B^®^), Department of Biomedical Engineering, Texas A&M University, College Station, TX, United States; ^4^ Department of Cardiovascular Sciences, Houston Methodist Institute for Academic Medicine and Full Affiliate Member, Houston Methodist Research Institute, Houston, TX, United States; ^5^ ABTECH Scientific, Inc., Biotechnology Research Park, Richmond, VA, United States

**Keywords:** theragnostics, cancer vaccine, RNA therapy, biomarkers, immunotherapy, personalized medicine, feedback control

## Abstract

Cancer continues to be one of the leading causes of death worldwide, and conventional cancer therapies such as chemotherapy, radiation therapy, and surgery have limitations. RNA therapy and cancer vaccines hold considerable promise as an alternative to conventional therapies for their ability to enable personalized therapy with improved efficacy and reduced side effects. The principal approach of cancer vaccines is to induce a specific immune response against cancer cells. However, a major challenge in cancer immunotherapy is to predict which patients will respond to treatment and to monitor the efficacy of the vaccine during treatment. Theragnostics, an integration of diagnostic and therapeutic capabilities into a single hybrid platform system, has the potential to address these challenges by enabling real-time monitoring of treatment response while allowing endogenously controlled personalized treatment adjustments. In this article, we review the current state-of-the-art in theragnostics for cancer vaccines and RNA therapy, including imaging agents, biomarkers, and other diagnostic tools relevant to cancer, and their application in cancer therapy development and personalization. We also discuss the opportunities and challenges for further development and clinical translation of theragnostics in cancer vaccines.

## Introduction

Cancer is a leading cause of death worldwide, with millions of new cases and deaths reported every year (Ferlay et al., 2021; Jacques Ferlay et al., 2020). Despite advances in conventional cancer treatments such as chemotherapy and radiation therapy, there is still a need for more effective and targeted therapies (Siegel et al., 2023). One promising approach is the use of cancer vaccines. Cancer vaccines aim to harness the patient’s immune system to target and destroy cancer cells (Galluzzi et al., 2017). Moreover, synergistic combinations of immunotherapy agents with conventional cancer treatments, offer yet another level of promise (Melero et al., 2015). Such is the case for resectable stage II–IV cutaneous squamous cell carcinoma (Howlader et al., 2020) and stage I–III non-small cell lung cancer (Forde et al., 2022). However, despite the long arc toward rational design (De Gregorio and Rappuoli, 2014), the development of cancer vaccines is complicated by the heterogeneity of tumors, the lack of effective immune response in some patients, and the difficulty in monitoring treatment response (Chuah and Chew, 2020). Tumor-based cancer vaccines were one of the initial steps to recruit the immune system in the fight against cancer (Kamath, 2021). Vaccines have several benefits over chemotherapeutic agents and monoclonal antibodies: for example, malignancy recurrence can be prevented by prolonged immunologic memories from the efficacious vaccination protecting against diverse cancer antigens. Additionally, vaccines do not need to be employed constantly and are relatively more secure than chemotherapy (Lollini et al., 2006). More often, vaccines are obtained by combination of the specific antigens like peptides, proteins, membranes, polysaccharides to induce controllable immune responses with synthetic or natural nanostructures/capsules to make vaccines more adjustable and safe. Different nanoparticles (NPs), including polymeric, inorganic, lipid- and protein/peptide-based, have been widely employed as adjuvants, immunogens, and antigen delivery vehicles for activating the innate immune system and that response is strongly influenced by NP’s size, shape, hydrophobicity and surface presenting chemistry (Liu et al., 2017). An additional approach is the use of RNA molecules to treat cancer and other diseases, which is an exciting concept with both inspiring potential and many challenges. Several genetic mutations manifest disease through the failure of cellular systems to produce properly functioning proteins (Alhmoud et al., 2020). RNA-based drugs can inhibit a variety of genes in multiple cellular pathways, target multi-gene diseases such as tumors, reduce drug resistance of tumor cells, and stop tumor proliferation. RNA therapy with high specificity, new targets and drug properties, demonstrate unique advantages in cancer therapeutics (Liang et al., 2020). Vaccines based on mRNA technology have had considerable success in addressing the spread, hospitalizations, and mortality during the COVID-19 pandemic. A key aspect of their efficacy is found in a formulation that uses lipid nanoparticles that enabled mRNA to enter the cell and initiate spike protein synthesis in the ribosomes. This formulation typically comprises four components, with particular emphasis on ionizable lipids and PEG lipids. Scale up and production of large quantities of high quality ionizable and PEG lipids, with attendant challenges in areas of purification and analysis, also challenged the production of large quantities of high-quality lipid nanoparticles.

Theragnostics, which seeks to combine diagnostic and therapeutic capabilities in a single platform system, have the potential to address these challenges by providing real-time monitoring of treatment response and enabling personalized treatment adjustments (Wang et al., 2021; Arnold, 2022). The sensing, measuring, and actively responding technical (SMART) platform systems enable closed-loop control of delivery, responding to therapeutic levels via a feedback mechanism. A wide range of nanomaterials feature quite prominently in imaging, drug delivery, and targeting within the tumor microenvironment (TME) and take advantage of the enhanced permeability and retention (EPR) effect and so are accordingly foundational in theragnostics (Cheng et al., 2021a).

In this article, we review the current state-of-the-science in theragnostics for cancer vaccines and RNA therapy, discuss the opportunities and challenges for further development and clinical translation. We used PubMed, Web of Science, and Google Scholar databases (2000–2023) to identify relevant studies on theragnostics in cancer vaccines and RNA therapy. As background, we introduce the elementary concepts of RNA therapy and the rudimentary concepts of engineering control models and their applicability to cancer theragnostics. The term theragnostics is justifiably used throughout this review (Frangos and Buscombe, 2019) despite the growing popularity of the use of theranostics, which is now being narrowly applied to radioligand imaging with therapy, i.e., precision oncology, which does not meet the broader definition of theragnostics as it lacks the four key elements of an active closed-loop control system. This review focuses exclusively on theragnostic approaches to cancer vaccines. We begin with an overview of cancer vaccines, RNA therapeutics and of control theory relevant for a discussion of closed-loop cancer theragnostics. We then present the enabling components for theragnostics in cancer vaccines; delivery vehicles and methods of theragnostic activation. We then show how RNA therapeutic agents may be creatively delivered and activated to achieve modulatable therapeutic levels. This is followed by presentation of a robust example of theragnostics for castration resistant prostate cancer. Finally, we provide our perspectives on opportunities and challenges, and future directions in the development of cancer theragnostics. It should be noted that this is not a comprehensive review of all relevant enabling aspects of cancer theragnostics, but rather to discuss some key progress in each area over the past several years.

## Overview of cancer vaccines

Cancer vaccines focus on therapeutic intervention in response to the disease and at thus unlike other vaccines that seek prevention as in the case of infectious disease (Antonarelli et al., 2021). Cancer vaccines can be broadly categorized into two types: prophylactic vaccines and therapeutic vaccines (Garbuglia et al., 2020). Prophylactic vaccines aim to prevent cancer by targeting cancer-causing viruses, such as the human papillomavirus (HPV) and hepatitis B virus (HBV). Since its 2006 approval by the USFDA, vaccines against cervical cancer-causing HPV-16 and HPV-18 have contributed to an overall reduction of 65% during the 2012 through 2019 period (Rosenblum et al., 2022). In contrast, therapeutic cancer vaccines aim to treat established cancers by stimulating the immune system sufficient to overcome immunosuppressive mechanisms employed by tumor cells, and so target and destroy cancer cells (Bilusic and Madan, 2012). Therapeutic vaccines can be based on various approaches, including tumor-associated antigens, cancer-specific mutations, or dendritic cells loaded with tumor antigens (Kerr et al., 2021).

One particularly notable path for therapeutic cancer vaccines is to induce antigen specific T-cell based cellular immunity capable of targeting and clearing tumor cells. These vaccines activate T-cell response against two types of tumor-specific antigens (TSAs), including viral antigens and neo-epitopes resulting from non-synonymous somatic mutations, and two types of tumor-associated antigens (TAAs), including tissue-specific antigens and development-specific antigens (Malekzadeh et al., 2020). There are typically three component signals to T-cell activation; the first is presentation of an epitope on a human leukocyte antigen (HLA) expressed by antigen presenting cells (APC), the second is co-stimulation by receptors on the APC, and finally, signaling by cytokines such as interleukin (IL)-12. Generation of CD4^+^ T cells is critical for the formation CD8^+^ T effector cells and CD8^+^ T memory cells during the antitumor immune response that is closely associated with antitumor immunity in many cancers (Angell and Galon, 2013; Bruni et al., 2020). However, tumor antigens cause weak CD4^+^ T cell help responses. Thus, cancer vaccines should combine efforts to engage more active CD4^+^ T cell directly or indirectly. Cancer vaccine immunotherapies such as immune checkpoint blockade (ICB) and T-cell receptor (TCR) or chimeric antigen receptor (CAR) T-cell adoptive therapy represent types of “vaccination” that raise antigen specific T-cell responses but without therapeutically administering antigen. The antigen is instead endogenously presented by the tumor itself (Smith et al., 2021).

Sipuleucel-T, an autologous cellular immunotherapy, is an example of autologous dendritic cell (DC) vaccine for the treatment of metastatic castrate-resistant prostate cancer, which was approved by the USFDA in 2010 (Sobol et al., 2015). It is generated by stimulation of the patient-derived DC to express a fusion protein consisting of the prostatic acid phosphatase (PAP) and granulocyte-macrophage colony-stimulating factor (GM-CSF). This vaccine therapy demonstrated a 4-month improvement in overall patient survival (OS). Unfortunately, despite this approval and OS benefit, this vaccine had many limitations in patient eligibility—lack of a pharmacodynamic biomarkers, high cost (nearly $100,000 for entire treatment course), and treatment inconvenience (requires leukapheresis followed by reinfusion with each cycle). Thus, the effect of Sipuleucel-T on patient health and therapy was very low indicating the need to improve these therapeutic approaches (Madan et al., 2020).

Despite the potential of cancer vaccines, their success is often limited by the complexity and heterogeneity of tumors. Tumors can evolve and adapt to evade immune recognition, and different patients may have different immune profiles that influence their response to the vaccine. In addition, the lack of effective biomarkers to monitor treatment response can make it difficult to determine the efficacy of the vaccine and to make informed decisions about adjustments to the treatment plan. As an example, the functional state of T-cell immunity is a key determinant in the success or failure of vaccination. Lifelong changes to the T-cell immune system, from immaturity to increasing senescence in later life, must accommodate periodic antigenic challenges from infectious agents such as viruses, bacteria, fungi, and allergens, each capable of causing acute, chronic, or latent infection and noninfectious transformation of cells, self-antigen, and allergens. T-cells must accurately interrogate and interpret each of these challenges and do so often in the context of some degree of immune suppression or inflammation.

An additional factor is the potential for development of the resistance to the cancer vaccines, which is based on the mutations in signaling pathways supporting tumor-immune control, downregulation or lost tumor antigen expression, altered antigen processing pathways, or loss of HLA expression and finally resulting in the low recognition of the tumor cells by T-cells (Saleh et al., 2020). A compounding challenge is so called T-cell exhaustion (Abdel-Hakeem et al., 2021; Yi et al., 2010), cellular dysfunction that emerges within hours that results in effector function impairment, compromising the ability of T-cells to effectively respond to new or renewed HLA challenges (Rudloff et al., 2023; Liu et al., 2021). Different mechanisms of tumor resistance to vaccines set the requirements to the multiple or temporal therapy approaches to mitigate or overcome resistance. Several strategies have been developed to solve tumor escape and tumor microenvironment immunosuppression, including improving immunotherapy delivery platforms/antigen selection, combination therapy and theragnostics.

## Overview of RNA therapy

**FIGURE 1:**
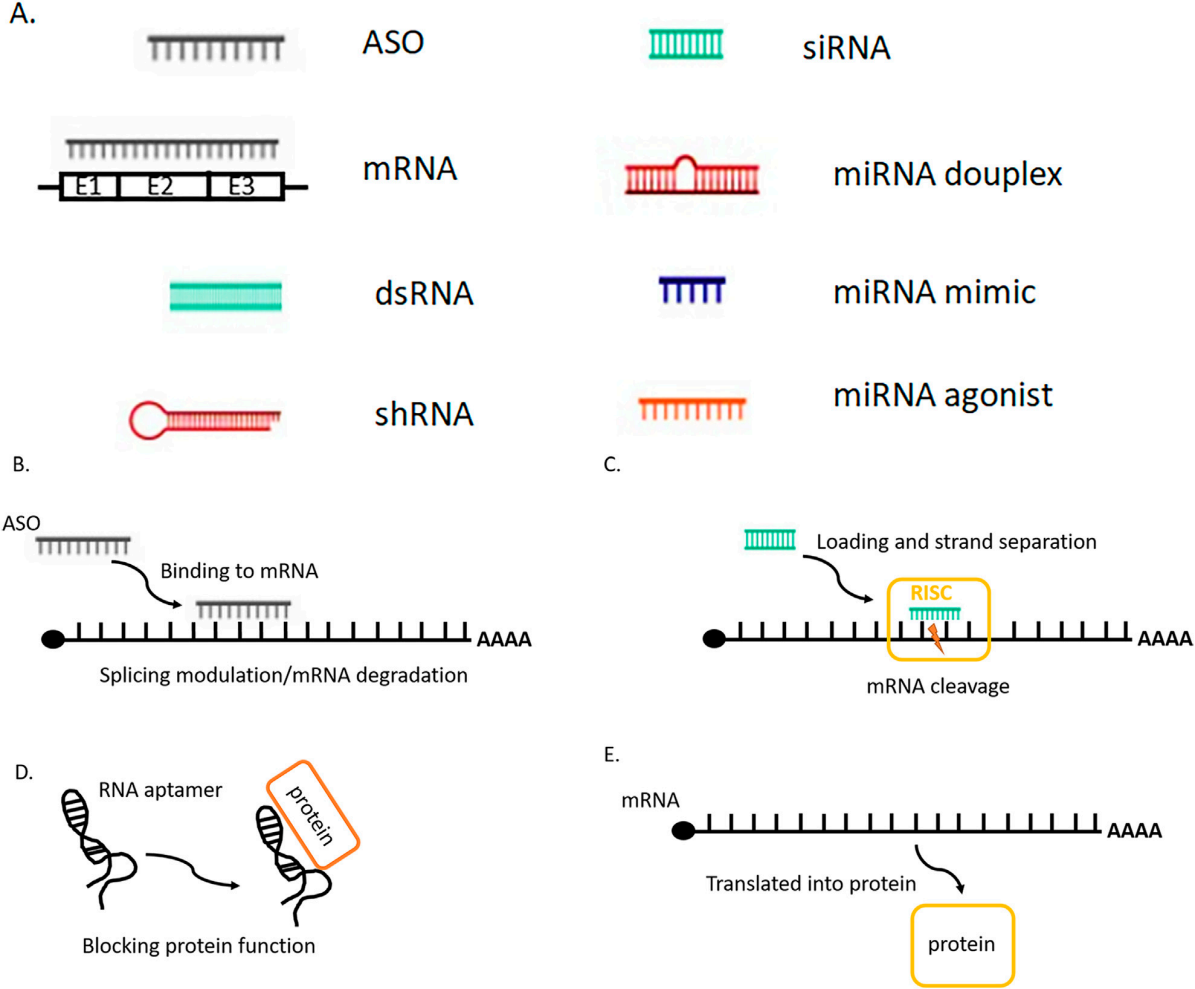
**(A)** Sсhematic visualization of the main RNA therapy molecules. **(B)** Antisense RNA (ASO) is designed to bind to pre- or mature mRNA and then induces the degradation of mRNA or modulates the splicing of pre-mRNA. **(C)** Small interfering RNA (siRNA) is introduced as a double-stranded form, after the loading into the RNA-induced silencing complex (RISC) one strand is removed. The siRNA-RISC complex binds to target mRNA and cleaves the mRNA inducing its degradation. Similar mechanism works for shRNA, miRNA duplex. **(D)** The RNA aptamer can bind to a specific protein and block its function. **(E)** After the messenger RNA (mRNA) is introduced into the cells, cellular machinery including the ribosome translates it into a protein, which works as an enzyme or antigen.

In the past few years RNA therapy approach has made a huge clinical impact, more than 13 drugs were approved by FDA from 2013. RNA drugs are often classified by the biochemical mechanism of action used to manipulate genes or gene expression. Accumulation of various types of gene mutations and incorrect regulation of gene networks formed by the interaction of these mutated genes are the main reasons for cancer development. Fundamental treatment approach is based on gene therapy. RNA therapy includes small interfering RNA (siRNA), antisense oligonucleotides (ASO), aptamers, mRNA and other RNA molecules (Figure 1), which can modulate the expression of target genes by different mechanisms of action with low side effects and low risk, blocking the inherent immunosuppression and triggering immune attacks on tumors (Kim, 2020; Ferdows et al., 2022). Small-molecule therapeutics is limited by the affinity to the target protein, RNA interference (RNAi) therapies (siRNA) modulate the protein translation. Similarly, antisense oligonucleotides (ASOs) have been used in the clinic to promote RNA degradation, such as inotersen (Benson et al., 2018), or to manipulate RNA splicing, such as eteplirsen (Kinali et al., 2009). Moreover, one of the advantages of RNA therapy is the rapid development of efficacious and targeted drugs for controlling tumor growth and regulation by that which is “undruggable” by small molecules and protein targets (Mansoori et al., 2014). The application of RNA therapy in cancer is mainly revealed in the following generalized approaches: 1) inhibition of tumor anti-apoptosis genes, 2) study of tumor signal transduction pathways, 3) inhibition of tumor angiogenesis-related factors, 4) the effect on oncogenes, 5) tumor suppressor genes, 6) reduction of tumor drug resistance, and 7) immunotherapy.

The application of RNAi in cancer therapy is mainly applied in its ability to suppress the anti-apoptotic genes, angiogenesis factors genes, and reduction of tumor drug resistance. Many studies have demonstrated the potential of siRNA delivery to the tumor site. For example, siRNA can be efficiently delivered into cancer cells and specifically inhibit the expression of anti-apoptosis genes, such as Bcl-2, Bcl-xl, XIAP (Kunze et al., 2012), as well as the genes encoding endothelial growth factor receptors (VEGFR 2 and EGFR), triggering the cell apoptosis and simultaneously improving the sensitivity of cancer cells to chemotherapeutic drugs. mRNA vaccine immunotherapy is a relatively new approach focused on the development of personalized mRNA vaccines for the treatment of various cancers. Broadly, nucleic acid cancer vaccines contain antigens encoded by either DNA or RNA and can be further subdivided into RNA and DNA vaccines that utilize different mechanisms for therapeutic delivery. But the main limitations of RNA therapeutics for the cancer treatment are poor stability of oligonucleotides in blood, low delivery efficiency, rapid renal clearance, and potential systemic toxicity (Li et al., 2024). To overcome these limitations, many RNA therapeutics delivery approaches have been proposed to improve the therapeutic efficacy of tumors.

## Overview of control theory and models in cancer

**FIGURE 2:**
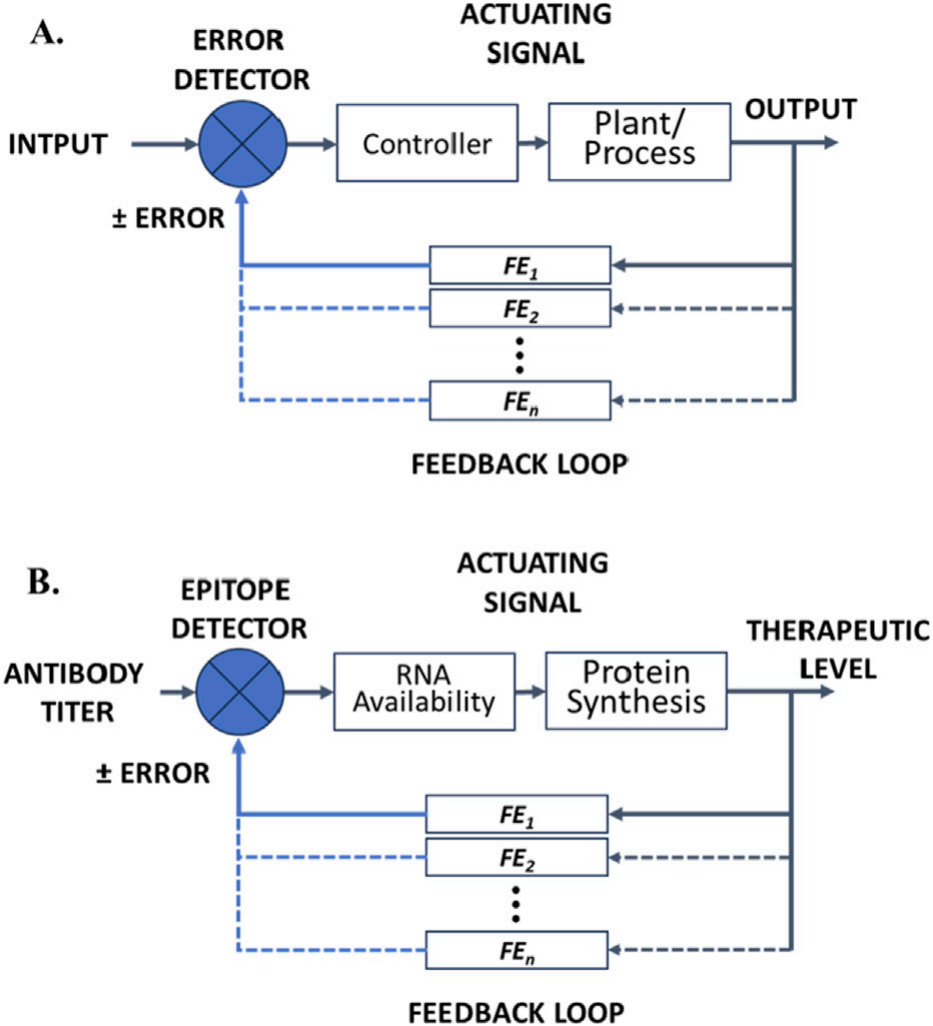
**(A)** Block flow diagram illustrating the key components of a closed-loop control system showing multiple possible feedback elements (*FE*_*1*_ *•••FE*_*n*_). **(B)** Sсhematic illustration of a hypothetical RNA theragnostic nano-delivery platform for cancer vaccines and RNA therapeutic drugs. The theragnostic platform shows different therapeutic levels achieved (output) that serve to adjust input via a series of feedback effects that affect the bioavailablity of RNA for any of the various mechanisms summarized in Figure 1.

Theragnostic platforms that are designed to achieve a particular level of therapeutic control must employ feedback control and are thus subject to the fundamental concepts and principles for understanding and designing engineered control systems (Iglesias and Ingalls, 2010). A control system manages, directs, or regulates the behavior of another system or process to achieve desired goals or performance (LeDuc et al., 2011). In the case of cancer, this is not just targeted delivery of a drug, but targeted therapeutic levels in response to the drug. Control theory underlies the control of dynamic systems, ensuring desired, predictable and stable behavior or performance based on pragmatic models. Such systems (models) may be open-loop, clinician-in-the-loop, or closed-loop systems. Figure 2A provides a schematic illustration of a generalized control system showing the key components and multiple feedback elements.

### Control systems or models

An open loop system does not employ feedback to control the output or therapeutic level. Such a drug delivery system operates solely based on the delivered drug and the existing pathophysiology, which serves as a set of preset conditions without regard to monitoring or adjusting based on the desired or achieved therapeutic level. Such drug delivery systems are simple, inexpensive to develop and easy to deploy (relatively) as they do not require an integral sensor to monitor the actual therapeutic level achieved and a validated feedback mechanism. Such systems are inherently inaccurate (may not achieve targeted therapeutic levels), imprecise (e.g., variability arising from polymorphisms. i.e., not personalizable) and unreliable (potential for failing to achieve targeted therapeutic levels) when deployed on their own.

A clinician-in-the-loop system is a hybrid approach that incorporates a human operator into the control loop, blending aspects of both open loop and closed loop control (next). Immortalized by the cultural cliché “take two pills and call me in the morning,” the human operator plays a critical role in the decision-making process, monitoring, and taking control actions, while the automated components execute tasks based on both the operator’s inputs, e.g., predefined dosing, targeting and designed-in drug release characteristics. This approach leverages the strengths of both human intuition, judgment, and adaptability and the precision, speed, and reliability of automated systems. The clinician makes decisions regarding manual inputs, e.g., dose and frequency of dosing, may override automatic controls, or adjust parameters in real-time based on situational awareness. This establishes flexibility in the control environment that can adapt to complex or unpredictable situations where automated systems alone might not suffice. The clinician receives feedback (MRI images, immunology titers, etc.) about the patient’s state and therapeutic performance, which they use to make informed decisions or make adjustments. The effectiveness of the clinician-in-the-loop control system depends on the seamless interaction between the human operator and the automated components, requiring well-designed interfaces and communication protocols. Being asynchronous, clinician-in-the-loop systems are prone to the delays incurred by the clinician schedule, clinician workload and error, and by interfaces, human and technical, that hinder rather than enhance clinician performance and decision-making. This is the basis for today’s theranostics.

Closed loop or feedback control systems use feedback to adjust the system’s output. Integrated sensors monitor the output and make adjustments to maintain the desired therapeutic level, even in the face of disturbances. Such systems are complicated, costly to develop, and costly to implement, in part because of the need for additional molecular recognition components for sensing and feedback via modulatable delivery or modulatable activity of the delivered therapeutic. However, closed loop systems are generally more accurate and reliable than open loop systems. They are intended to adapt to changes in the environment and correct for perturbations/disturbances, while maintaining consistent performance and hence form the basis for highly personalized therapy. This is the premise for and promise of theragnostics. Figure 2B provides a schematic illustration of a generalized control system showing the key components and multiple feedback elements when applied to RNA cancer theragnostics.

### Elementary control theory for cancer theragnostics

Control systems consist of the following key elements: i) an input, a reference or setpoint value, that is a target that the control system aims to achieve, ii) the process which the control system seeks to control, iii) a sensor that assess the present state or output of the process and provides feedback to the control system about the actual performance of the process, iv) a controller that processes the feedback information from the sensor and computes the control action necessary to adjust the process to match the desired setpoint, and v) an actuator that executes the control action to bring the controlled system closer to the desired state. In addition to the forgoing, there are: vi) the control signal, which is the output from the controller to the actuator, intended to produce the corrective action, vii) a feedback loop that links the sensor, controller, and actuator, creating an open-loop control (the control input is determined without considering feedback from the system’s output) or a closed-loop control (feedback is used to adjust the control input based on the system’s response) that allows for continual adjustments based on the measured output, viii) the reference or setpoint that serves as the target value that the control system is seeking to attain. The controller uses the difference between the setpoint (e.g., a targeted antibody titer) and the measured output (the actual antibody titer) (the error) to generate the control signal, ix) the mode of response of the controller may be proportional (P) (Equation 1), integrative (I) (Equation 2), or derivative (D) (Equation 3) as shown following.

$$Propotional\;control\;response=f\left(error\right)$$ (1)

$$Integrative\;control\;response=f\left(\int_{t_{1}}^{t_{2}}\left(error\right)\right)$$ (2)

$$Derivative\;control\;response=f\left(\frac{dx}{dt}\left(error\right)\right)$$ (3)

In the proportional mode, the controller’s output is directly proportional to the error signal, being based solely on that value, without regard to past or future errors. This may be represented by Equation 4:

$$P=K_{p}\times e\left(t\right)$$ (4)

Where P is the output of the proportional controller, $K_{p}$ is the proportional gain, a constant that determines the sensitivity of the controller to the error signal and $e\left(t\right)$ is the error signal at time t . Such systems are quick to respond but are prone to steady-state errors, failing to reach the desired setpoint. In the integral mode, the controller’s output is proportional to both the magnitude and the duration of the error signal over time. The controller continuously sums up the error signal over time, adjusting its output accordingly. This may be represented by Equation 5:

$$I=K_{i}\times \int_{t_{1}}^{t_{2}}e\left(t\right)\;dt$$ (5)

Where I is the output of the integral controller, $K_{i}$ is the integral gain, a proportionality that establishes how aggressively the controller responds to the accumulated error, $e\left(t\right)$ is the error signal at time t and the integral represents the accumulated contribution of the error signal over time. This approach eliminates the steady-state error of the proportional mode but is slow to update, and depending upon the value of the integral gain, may overshoot the targeted therapeutic level, seek to correct and produce dynamic instability. In the derivative mode, the controller’s output is proportional to the rate of change of the error signal. It predicts the future behavior of the error signal based on its present rate of change. This may be represented by Equation 6:

$$D=K_{d}\;\frac{d\left[e\left(t\right)\right]}{dt}$$ (6)

Where D is the output of the derivative controller, $K_{d}$ is the derivative gain, controlling the sensitivity of the controller to the rate of change of the error, and $\frac{d\left[e\left(t\right)\right]}{dt}$ is the derivative of the error signal with respect to time. Such a controller dampens oscillations and stabilizes the system by predicting and responding to rapid changes in the error signal. However, such a controller can amplify noise in the system, leading to instability and/or erratic behavior, especially in systems with high-frequency noise.

Finally, there is, x) the output of the process, the controlled variable, which represents the actual result or performance of the system being controlled. The control system aims to maintain this variable as close to the setpoint as possible. When applied to RNA therapy and cancer vaccines, such systems are generally called biologically responsive (bioresponsive) or biosmart and are exemplified by bioresponsive hydrogels (Wilson and Guiseppi-Elie, 2013). When engineered into bioresponsive, adaptive controlled delivery systems they are exemplified by the modulated release of insulin from a chemically synthesized artificial pancreas made responsive to glucose through the action of gluconic acid produced within a pH-responsive hydrogel (Guiseppi-Elie et al., 2002; Bhat et al., 2020). To evaluate the performance of molecular control systems, an engineer-centric view requires that we entertain consideration of such characteristics as rise time, settling time, overshoot, and steady-state error. A theoretical framework allows for modeling, analysis and optimization enabling the design of smart, adaptive, and precise theragnostic systems that can optimize therapeutic outcomes while minimizing side effects, making them especially valuable in personalized cancer care.

Control theory has been applied with varying levels of success to the optimization of chemotherapeutic agents [summarized in (Lecca, 2021)] and radiation [summarized in (Jarrett et al., 2020)] used in the treatment of solid tumors. A general formulation of time varying problems restricted to linear differential equations may be written when the state variables at time, *t*, are *x*_1_(*t*), *x*_2_(*t*), …, *x*_*n*_(*t*) and the system inputs at time, *t*, are *u*_1_(*t*), *u*_2_(*t*), …, *n*_*m*_(*t*). The system then can be represented by *n* differential equations, each varying in time, and represented as a matrix (Equation 7),

$$\frac{d\left[C_{c}\right]}{dt}=f\left(x\left(t\right),u\left(t\right),t\right)$$ (7)

where $C_{c}$ is a measurable and modulatable cancer characteristic such as a biomarker associated with efficacy of the therapy, *t*_*1*_ and *t*_*2*_ are time step 1 and time step 2, respectively and *dt* is an increment of time in the derivative. For solid tumors, the cancer characteristic may be the tumor volume, or more correctly, the surface to volume double integral. The rate of change of the cancer characteristic, $d\left[C_{c}\right]/dt$ , is a function (*f*) of chemotherapy dose, $x\left(t\right),$ and seeks to optimize, for example, the drug infusion schedule, $u\left(t\right),$ that most effectively reduces the size of the tumor following a fixed period of treatment. Next-generation theragnostics seek models for closed loop control by employing bio-smart delivery vehicles based on responsive nanoparticles. An example is the work of Annapragada’s group wherein liposomal nanoparticles were aggregated by the use of borate, when upon binding with glucose, disaggregates the nanoparticles allowing their release, loaded with insulin (Karathanasis et al., 2007; Dasgupta et al., 2012).

## Theragnostics in cancer vaccines

In oncology, tumor-specific substrates, receptor ligands, or pro-drugs can serve as constructs for theragnostic development when labeled with specific radionuclides for imaging or therapy. A cursory Google search (04/25/2024) reveals that theranostics (without the “g”) produced 42,900,000 results and theragnostics (with the “g”) produced 106,000 results. Theranostics has become a popular sub-field of nuclear medicine that co-joins imaging with *ex-vivo*, in-the-loop therapeutic intervention. An illustrative example is the use of ^68^Ga and ^177^Lu labeled peptides targeting fibroblast activation protein (FAP) and positron emission tomography (PET) in the development of cancer theranostics (Huang et al., 2023). FAP, a glycoprotein of the dipeptidyl peptidase family, is abundantly expressed in cancer-associated fibroblasts (CAFs) of numerous epithelial tumors (e.g., sarcoma and mesothelioma). Targeting and imaging this glycoprotein provides theranostic insight into the progression of disease (Huang et al., 2023). Theragnostics embodies the broader class of therapeutic agents, e.g., RNA therapy and cancer vaccines, as well as stimuli-responsive drug delivery systems (DDS) that are modulated by the desired level of an induced therapeutic protein or immune response.

**FIGURE 3:**
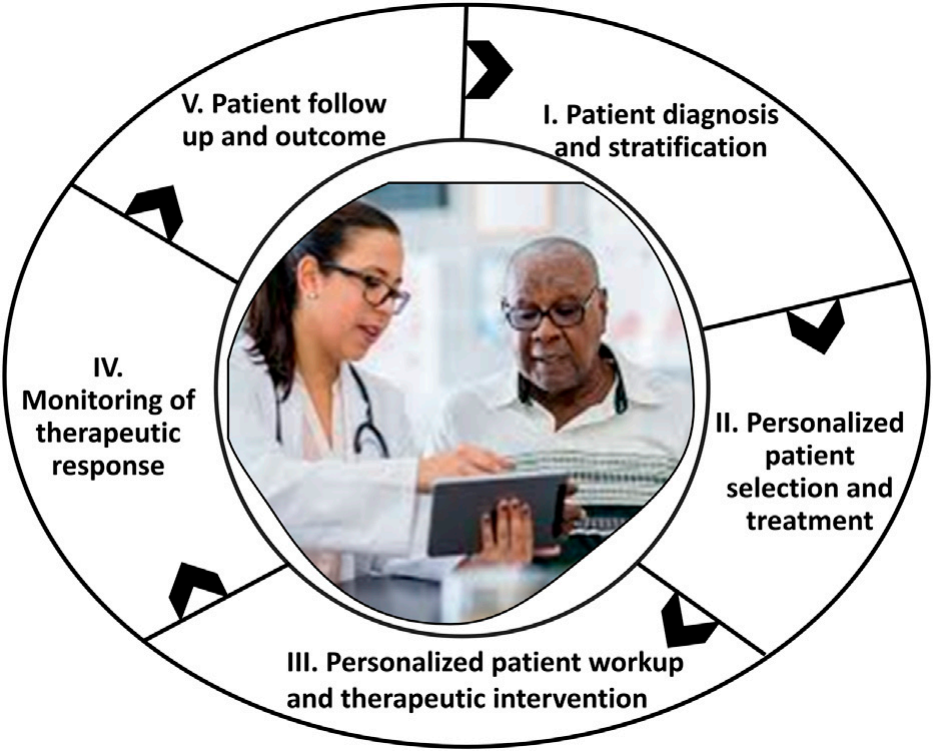
A generalized scheme for the outcomes-driven, patient-centered administration of cancer vaccine theragnostics.

Development of novel RNA-based therapeutic treatments for cancer and orphan diseases, including the development of mRNA vaccines and novel RNA-based therapeutic systems to target **and** modulate/potentiate gene expression and RNA-protein interactions forms one of the basis for cancer vaccine theragnostics (Zhu et al., 2022). One approach, reviewed by Guo et al. (2022), is to synthesize biomimetic nanoparticles conferred with the targeting and immune evasion qualities of cancer cell membranes (CCMs) (Guo et al., 2022). The development of novel strategies for targeting and modulating epigenetic pathways that are involved in the regulation of gene expression and tumorigenesis represents another approach (Cheng et al., 2019). Theragnostics offer a promising solution to the challenges of cancer vaccine development and personalization (Ferrari, 2005). Theragnostic agents combine both diagnostic and therapeutic capabilities in a single platform system, enabling real-time monitoring of treatment response and personalized treatment adjustments. A theragnostic approach accords the following (Figure 3):

I) Diagnosis, stratification, possibly using AI-based multi-modal data fusion, and patient selection.
II) Development of a personalized treatment plan.
II) Personalized patient workup and therapeutic intervention
III) Therapy response monitoring—visualizing where the drug is going and in turn monitoring the efficacy of the treatment. This allows the identification of likely responders leading to faster and cost-efficient clinical trials and increased chances of successful treatment outcomes.
IV) Personalized dosing based on individual imaging data, thus achieving maximal therapeutic effect with minimal unwanted side effects.
V) Patient follow up and survival outcomes (SO) monitoring.

The functional linking of the cancer diagnostic with the therapeutic intervention is what uniquely characterizes a theragnostic system. One approach to theragnostics in cancer vaccines is the use of imaging agents to monitor the migration of immune cells to the tumor site. For example, PET imaging can be used to track the accumulation of immune cells at the tumor site, and MRI can provide information about tumor volume and vasculature. In addition, biomarkers such as circulating tumor cells, circulating tumor DNA, or immune cell profiling can provide valuable information about the patient’s immune response to the vaccine.

Another approach to theragnostics in cancer vaccines is the use of personalized antigen selection based on tumor-specific mutations (Blass and Ott, 2021). The identification of specific mutations in the tumor can guide the selection of antigen targets for the vaccine, enabling a more personalized approach to therapy (Lin et al., 2022). Use of tumor-associated antigens (TAAs) and tumor-specific antigens (TSAs) to activate the patient’s immune system, can in principle, induce both specific cellular immunity and humoral immune response to prevent tumor growth and ultimately eradicate tumor cells (Jhunjhunwala et al., 2021; Zhao et al., 2021a).

New and perspective approach to theragnostics based on DNA or mRNA cancer vaccines continue to emerge. In 2023 more than 35 clinical trials were evaluating the safety and efficacy of mRNA cancer vaccines for select cancer types. For example, the embryonic stem cell gene SRY (sex determining region Y)-box 2 (SOX2) is an oncogenic driver in non-small-cell lung cancer and the basis of the promising DNA cancer vaccine coded the fusion protein with the PADRE helper epitope. In mice, a SOX2 vaccine inhibited the growth of the TC-1 lung cancer cell line characterized by high SOX2 production. Both humoral immune responses and T-cell responses against SOX2 correlates with clinical response in patients receiving immunotherapy (Polakova et al., 2014; Spisek et al., 2007). Also, the use of theragnostic agents can help to identify patients who are likely to respond to immunotherapy, and to monitor their response over time.

Many years ago the importance of the critical relationship between the immune system and radiation therapy was demonstrated. Possibly, the radiation therapy can provoke a tumor-specific immune response that not only targets cancer cells locally but can also travel to distant sites of disease and act as an *in situ* vaccine, resulting in a systemic response. Additional evidence for this proposal is supported by the synergistic effects of radiation and immunotherapies, which have demonstrated improved clinical response, overall survival, and time to recurrence in multiple cancer histologies (Formenti and Demaria, 2012; Tang et al., 2014). mRNA cancer vaccines may encode the immunostimulants proteins, which can modify the tumor microenvironment and thus enhance the efficacy of the main therapy, or serve as an additional component for the diagnostic agent with the therapeutic action. Intratumorally administered mRNA-2416 produced by Moderna and encoded OX40L, demonstrated safety and tolerability and revealed proinflammatory activity with desirable changes in non-small cell lung cancer (Porciuncula et al, 2021). Several other mRNA products have also shown promise, such as ECI-006, a combination of TriMix and melanoma-specific TAAs administered intranodularly and being tested in a phase 1 study of resected melanoma (NCT03394937); and MEDI1191, an immunomodulatory fusion protein containing IL-12α and IL-12β subunits developed for intratumoral injection (Wang et al., 2023).

## Theragnostic delivery vehicles

**FIGURE 4:**
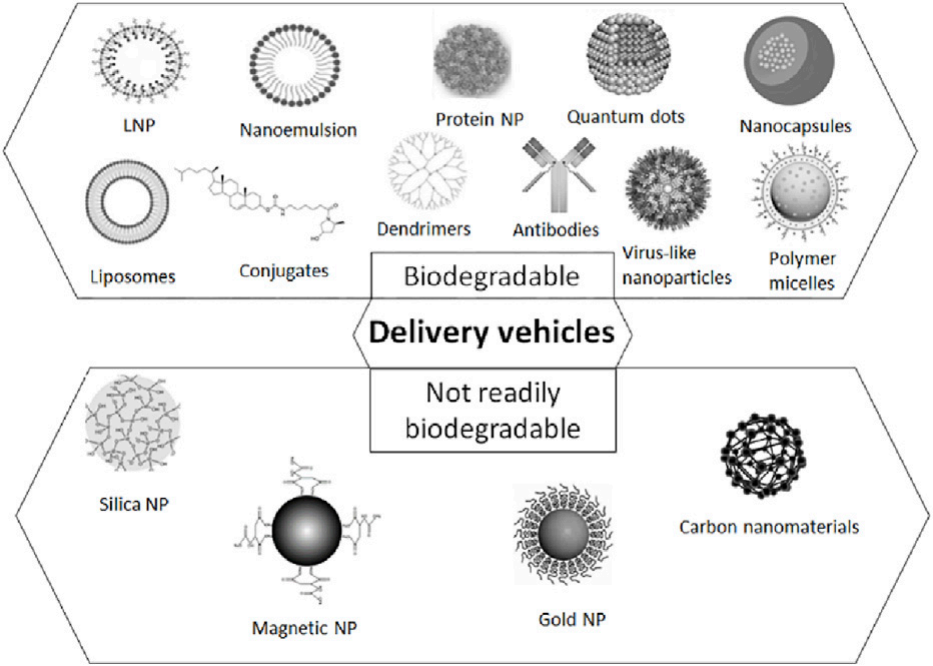
A schematic representation of the delivery platforms for cancer vaccine theragnostics, including polymeric NPs, silica- and carbon materials, virus-like NPs, inorganic NPs, lipid-based NPs, ligands and antibodies for the targeted delivery.

The integrative challenge of targeted delivery, response to biomarker signaling threshold, and delivery of payload places unique challenges on the biophysical and biochemical properties of delivery platforms for cancer theragnostics. In this section, we review possible delivery platforms that may be engineered for cancer vaccine theragnostics (Figure 4).

### Targeted drug delivery

Theragnostic delivery vehicles must be delivered to targeted cells within cancerous tissues. Targeted delivery of medications is an important aspect of modern drug delivery technology and also an instrument for the identification and validation of new therapeutic targets and increase of the therapeutic efficacy (Gao et al., 2023). The goal is to deliver medications to specific locations (e.g., sub-cellular organelles, cells, tissue types, organs) in the body, such as to the site of the disease, and to do so in a more efficient and effective manner than traditional systemic drug delivery methods. To overcome the barriers to safe and effective theragnostic drug delivery, viral-vector-based and non-viral delivery systems were developed. The main function is the protection of RNA from degradation, maximize delivery to on-target cells and minimize the off-target effects. Viral gene therapies have generated successful clinical cases but the efficacy was limited by pre-existing immunity, viral-induced immunogenicity (Bryson et al., 2017), unpredictable genomic integration and some difficulties with its production. In parallel, development of synthetic carriers that encapsulate RNA, such as polymers, lipids and lipid nanoparticles (LNPs), ligands, antibodies, liposomes, nanoparticles (NP), and microspheres, which are designed to improve the accuracy and efficiency of drug delivery (Adepu and Ramakrishna, 2021; Tewabe et al., 2021), has led to US Food and Drug Administration (FDA) approval of new therapeutics. The most revolutionary variants of drugs were subcutaneously administered N-acetylgalactosamine (GalNAc)–siRNA conjugates that target hepatocytes (Garrelfs et al., 2021), intravenously administered LNP-based siRNA drugs that target hepatocytes (Adams et al., 2018), and intramuscularly administered LNP-based mRNA COVID vaccines (Baden et al., 2020). Nanoparticle-based drug delivery systems may also be useful for non-viral DNA delivery (Buck et al., 2019). Additional efforts were spent to make the synthetic delivery vehicles biodegradable: inherently biodegradable is defined as > 20% but <60% biodegradability as measured by OECD 301A-F testing, readily biodegradable—the ability of a product to biodegrade quickly and completely (≥60% by OECD 301A-F/ASTM D7373 testing) within 28 days.

### Responsive drug delivery

Theragnostic delivery vehicles, once delivered to targeted cells, must respond to stimuli that enables release or activation of the RNA payload. Stimuli-responsive materials undergo a physicochemical (Kocak et al., 2017) or electrical (Samanta et al., 2016) change in response to an external stimulus. Such materials are great candidates for responsive drug delivery platforms that seek to alter drug release profile characteristics in response to the unique physiological condition or activity of a particular biomarker at the site of targeted tissue (Rahim et al., 2021; Sun and Davis, 2021). Bioactive and bioresponsive hydrogels (Wilson and Guiseppi-Elie, 2013) may be suitable candidates for cancer theragnostic platforms. These SMART nanogels possess engineered properties that enable sense and release under feedback control. First described by Kim and Park in 2001, these authors exploited the gel–sol phase transition of a membrane-supported, glucose-sensitive hydrogel composed of PEGylated-Con A and glucose-containing polymers. The gel–sol phase transition was titratable in response to the environmental glucose concentration over the range 1–4 mg/dL glucose (Kim and Park, 2001). In 2002, Guiseppi-Elie et al. (2002) engineered a self-contained, bioresponsive, adaptive controlled delivery system that was exemplified by the modulated release of insulin from a chemically synthesized artificial pancreas made responsive to glucose through the action of gluconic acid produced within a pH-responsive hydrogel (Bhat et al., 2020). Recently, there is renewed interest in stimuli-responsive targeted drug delivery systems (Li et al., 2019) (Majumder and Minko, 2021; Huang et al., 2019; Huang et al., 2021). Such systems are multi-functional, seeking to combine targeting with an endogenous or exogenous stimulus to effect release of the pro-drug or drug payload. Multi-functional systems of this type may be ON-OFF, with release occurring in response to a threshold amount of the stimuli, or may be potentiated, that is, the amount of drug released or the rate at which the drug is released is dependent on the magnitude of the stimuli (e.g., chemical potential of an effector molecule). A recent example is a hydrogel based on the L-arginine (L-Arg)-coupled chitosan and glucose oxidase (GOx)-modified hyaluronic acid, which in the presence of elevated levels of glucose, continuously released hydrogen peroxide (H_2_O_2_) and NO by the cascaded consumption of glucose and L-Arg that was shown to be an effective antibacterial *in vitro*, as well as *in vivo* wound healing performance on an infected diabetic mice model (Zhou et al., 2023). This concept is readily applied to cancer therapeutics, and eventually to cancer theragnostics.

Liu et al. (2023) exploited the high intracellular glutathione (GSH) levels in tumor tissues (2–10 mmol/L) to trigger the responsive release of lenalidomide from the redox-responsive prodrug of disulfide-lenalidomide-methoxy polyethylene glycol (LND-DSDA-mPEG). When combined with methotrexate (MTX), the resulting LND and MTX nanoparticles (MTX@LND NPs) were delivered via subcutaneous administration at the neck near the deep cervical lymph nodes (dCLNs) and were shown to inhibit the growth of lymphoma and effectively prevent liver metastasis (Liu et al., 2023). A nanocomplex (50 nm; PDI = 0.148) of anti-programmed death ligand-1 peptide (APP), spermidine (SPM) and oxidized dextran (DEXo) expanded with sodium tripolyphosphate (TPP) and FeCl_3_ was shown to have an inhibitory influence on lymphoma cells (A20) both *in vitro* and *in vivo* (mouse model). Spermidine, is a known regulator of the tumor microenvironment (TME) through its depletion of immunosuppressive cells and thereby attenuates immune surveillance in the TME. Iron-induced ferroptosis in cancer cells may trigger the release of immune-stimulative signals that facilitate the recruitment of dendritic cells (DCs), macrophages, or the other immune cells. The strong oxidative stress, consequent mitochondrial dysfunction and subsequent ferroptosis demonstrates a form of pH-responsive, multimodal therapy (Nie et al., 2023). The foregoing are representative examples of responsive systems that take advantage of the unique attributes of the TME. However, none rises to the level of responding to an output of the therapy that then becomes a setpoint for the control of the therapeutic intervention.

### Chemical conjugates

Drug conjugates are therapeutic agents formed from the physicochemical combination, predominantly covalent, of two or more actives intended to combine the pharmacological properties of their individual components to achieve specific therapeutic goals (Fu et al., 2022). The pharmacological reasons for synthesizing drug conjugates are as diverse as the conjugates themselves and depend on the specific therapeutic goals and challenges associated with a particular disease or condition being targeted. These conjugates offer a versatile approach to drug development and can address many limitations associated with traditional drug therapies. There are several pharmacological reasons to synthesize drug conjugates: i) Enhanced efficacy—drug conjugates can enhance the overall therapeutic efficacy of a drug by combining two or more mechanisms of action. For example, combining a cytotoxic drug with a targeting molecule can increase the drug’s specificity for cancer cells, reducing off-target effects and improving efficacy, ii) improved targeting—conjugates can be designed to target specific tissues, cells, or molecular markers. This targeted delivery can increase drug concentration at the desired site of action, minimizing exposure to healthy tissues and reducing side effects, iii) Overcoming drug resistance—drug resistance is a common problem in chemotherapy and other treatments. Drug conjugates can help overcome resistance by using alternative pathways or mechanisms to deliver the therapeutic payload to the target site, iv) controlled release-conjugates can be engineered to release the active drug at a controlled rate or in response to specific physiological conditions. This controlled release can optimize drug delivery and minimize toxicity, v) reduced toxicity—by selectively delivering drugs to their targeted cells or tissues, some drug conjugates can reduce the systemic exposure of healthy tissues to the toxic side effects of some drugs, thereby minimizing adverse effects, vi) increased solubility—some drugs have poor aqueous solubility, which can limit their effectiveness; conjugation with solubilizing agents can improve drug solubility and hence bioavailability, vii) prolonged half-life/protection from degradation—conjugation with certain molecules, such as PEG, can extend the circulating half-life of a drug in the body. This extended circulation time can reduce the frequency of dosing, viii) combination therapy—drug conjugates can be designed to deliver multiple drugs simultaneously, allowing for combination therapy, with synergistic effects or drugs that target different aspects of a disease (Zhao et al., 2023) ix) diagnostic applications—conjugates can also be used for diagnostic purposes. For example, radiolabeled antibodies can be used in imaging techniques like PET to detect specific disease markers, x) personalized medicine—drug conjugates can be customized based on a patient’s unique disease profile, allowing for more personalized and targeted treatments, xi) reduced immunogenicity and off-targets—conjugating drugs with certain molecules can reduce their immunogenicity, making them less likely to trigger an immune response, xii) improved pharmacokinetics—conjugation can alter the pharmacokinetic properties of drugs, such as biodistribution, metabolism, and excretion, to optimize their therapeutic profiles. While conjugation may be necessary, it cannot by itself comprise a theragnostic platform.

Conjugation with polymers or specific ligands is widely used for the enhanced delivery of therapeutic RNAs and theragnostic molecules. Direct covalent conjugation of various moieties: lipids (for example, cholesterol that facilitates interactions with lipoprotein particles) (Wolfrum et al., 2007; Lorenz et al., 2004), peptides (Eguchi et al., 2009; Liu et al., 2014), aptamers (McNamara et al., 2006), antibodies (Song et al., 2005) and sugars (for example, *N*-acetylgalactosamine (GalNAc) (Matsuda et al., 2015), promoted intracellular uptake, targeted the drug to specific cells/tissues or reduced clearance from circulation. Cholesterol conjugated siRNAs was applied for hepatic gene silencing (for example, Apolipoprotein B, Apob) (Soutschek et al., 2004) and, more recently, to silence myostatin (Mstn) in murine skeletal muscle (Khan et al., 2016). Docosahexaenoic acid (DHA), the most abundant polyunsaturated fatty acid in the mammalian brain, has been used as a conjugate to enhance siRNA delivery to the murine brain (Nikan et al., 2016). The most widely clinically validated example of a specific ligand is GalNAc conjugates with RNA therapeutic molecules, which have led to the FDA-approved drugs givosiran (Syed, 2021) and lumasiran (Garrelfs et al., 2021), as well as the EMA-approved drug inclisiran (Lamb, 2021). GalNAc is a carbohydrate-derived trivalent ligand that binds the asialoglycoprotein receptor, which is expressed in hepatocytes and not expressed on other cell types. Conjugation of theragnostic agents with antibodies or antibody fragments leads to extrahepatic delivery, for example, conjugates of siRNA with anti-CD71 antibody fragment had predominantly heart and skeletal distribution (Sugo et al., 2016). Whereas PEG, an FDA-approved polymer, was conjugated with the first RNA aptamer drug, Macugen (now discontinued), approved by FDA (Ng et al., 2006). Many investigations have demonstrated that hydrophobic conjugates accumulate in the liver, whereas less hydrophobic conjugates accumulate in the kidneys. Dichloroacetic acid and dichloroacetic acid with a phosphocholine polar head group improved siRNA delivery to extrahepatic tissues such as the lung and heart and, to a lesser degree, to skeletal muscle and fat in comparison to the cholesterol, which is a well-studied hepatic conjugate (Biscans et al., 2018).

### Nanoparticles used in cancer vaccines

Nanoparticles have been extensively studied and employed as cancer vaccine delivery vehicles (Wen et al., 2019). Several hold high promise with potential to serve as theragnostic platforms. For a comprehensive review of engineered nanoparticles with a focus on drug delivery see (Mitchell et al., 2021). These nanoparticles can serve various purposes, including targeting, drug payload delivery, diagnostic imaging, and immunomodulation.

The range of nanoparticles with potential for use in cancer vaccine theragnostics includes:

Lipid nanoparticles (LNPs) composed of natural and synthetic phospholipids, have gained attention for delivering mRNA-based cancer vaccines, such as those used in the development of mRNA COVID-19 vaccines of Pfizer-BioNTech and Moderna (Wilson and Geetha, 2022; Tenchov et al., 2021). LNPs are effective carriers of genetic material, like mRNA ASO, siRNAs, to target cells (Schoenmaker et al., 2021). FDA-approved LNPs contain variations of four basic components: a cationic or ionizable lipid, cholesterol, a helper lipid, and a poly (ethylene glycol) (PEG)-lipid. Typically fashioned from cationic lipids such as 1,2-dioleoyl-3-trimethylammonium-propane (DOTAP) and 1,2-dimyristoyl-sn-glycero-3-ethylphosphocholine (EPC) that electrostatically interact with the negatively charged nucleic acids to form stable complexes. Neutral lipids such as 1,2-Distearoyl-sn-glycero-3-phosphocholine (DSPC) and 1,2-Dioleoyl-sn-glycero-3-phosphocholine (DOPC) often form the core of the LNP and may be solid or liquid at physiological temperature. Supportive lipids such as cholesterol and/or PEGylated lipids are often included to enhance the rigidity and stability of the lipid bilayer to improve the stability, fusion capacity, and encapsulation efficiency and extend LNP circulation time. The main lipids for siRNA delivery included C12-200, cKK-E12, a peptide-like lipid compound, DLin-KC2-DMA, an ionizable lipid identified using rational design and DLin-MC3-DMA123, which was used in patisiran to treat hATTR1 (Adams et al., 2018). LNPs composed from cKK-E12124,125, C12-200126, and DLin-MC3-DMA127 applied for the mRNA delivery to the liver. Two LNPs formulated with an unreported cationic or ionizable lipid, PEG-lipid, cholesterol and 1,2-distearoyl-sn-glycero-3-phosphocholine (DSPC) used for the delivery of mRNA encoding a nickase Cas9 and sgRNA targeting PCSK9 to the liver in the primates (Paunovska et al., 2022).

LNPs protect the genetic material from *in vivo* sources of degradation and facilitate its uptake by cells. LNPs can also be engineered to carry both the therapeutic payload and serve as diagnostic components when functionalized for imaging. This allows for real-time monitoring of the vaccine’s effectiveness as well as the patient’s response. LNPs are a fixture in modern vaccine development and offer exciting prospects for personalized medicine and theragnostics by enabling targeted delivery, monitoring, and enhanced immune responses in the context of vaccination LNPs typically consist of lipids, cholesterol, a processing surfactant, and solvent in property determining ratio. These lipids form the 3-D structure of the nanoparticle and play a crucial role influencing particle size, surface charge, and stability. Commonly prepared by thin-film hydration, solvent displacement, or microfluidic techniques, LNPs are loaded, homogenized, and purified using example techniques of ultracentrifugation, size-exclusion chromatography, or dialysis (Duan et al., 2020; Roces et al., 2020).

Among the negative features of mRNA are i) the potential for an unintended immune response, with the body recognizing the mRNA or its delivery system (e.g., LNP) as foreign, leading to an inflammatory response, ii) mRNA molecules are inherently unstable, being readily degraded by RNases and thus necessitates low temperature storage and transportation, with costly and complicated logistics, iii) despite its success, efficient delivery of lipid-protected mRNA into the target cells without degradation is challenging (e.g., the distribution of mRNA among all LNPs), iv) the effects of mRNA therapy might be relatively short-lived compared to other types of treatments like DNA-based therapies, necessitating repeated dosing to maintain therapeutic benefits, thereby increasing the cost and complexity of treatment.

Liposomes comprising lipids, including phospholipids (e.g., phosphatidylcholine) and cholesterol, which form the characteristic bi- or more layer 3-D structure, are a specific sub-class of lipid-based nanoparticles that can encapsulate drugs or antigens for targeted delivery (Nel et al., 2023). Fashioned similar to LNP from phosphatidylcholines such as 1,2-dioleoyl-sn-glycero-3-phosphocholine (DOPC) and 1,2-distearoyl-sn-glycero-3-phosphocholine (DSPC) that form the main structural components of liposomes, they enable the unique bilayer structure and stability to the liposome. Cholesterol, a key component in many liposome formulations, is often included to enhance the stability and rigidity of the liposomal membrane. The cationic lipids, 1,2-dioleoyl-3-trimethylammonium-propane (DOTAP) and N-[1-(2,3-dioleoyloxy)propyl]-N,N,N-trimethylammonium chloride (DOTMA), may be included to provide electrostatic binding with the RNA payload. PEGylated lipids, fusogenic lipids, or lipids that enhance endosomal escape, such as ionizable lipids, confer buffering to the pH inside the endosome to facilitate endosomal escape of the payload into the cytoplasm. The oldest known nanoparticle drug delivery system, PEGylated liposome loaded with doxorubicin (DOX), was approved by the USFDA in 1995 for the treatment of AIDS-related Kaposi’s sarcoma (Barenholz, 2012) heralding in the era of nanomedicine. Lipids may be selected to promote specific properties like stability and fusogenicity. PEGylation, no longer favored because of the emergence of PEG antibody profiles (Yang et al., 2016; Chen et al., 2021), has resulted in more functionalities being introduced to the liposome platform, such as, *in vivo* imaging probes for optical, MRI, PET, and single-photon emission computed tomography (SPECT). The introduction of novel agents for photodynamic and photothermal therapies (PDT, PTT) serve to broaden the therapeutic potential of targeted delivery opportunities for liposomes. However, stimuli-responsive liposomes that possess pH, redox and temperature-sensitive lipid moieties engender the possibility for a theragnostic platform (Lee and Im, 2019).

Polymeric nanoparticles based on a variety synthetic, natural and hybrid biodegradable and biocompatible polymers like PLGA [poly (lactic-*co*-glycolic acid)], PEG, PEG-methacrylates, and Chitosan can be used to encapsulate antigens and adjuvants for controlled release and enhanced immune responses (Daramola et al., 2022). Polymeric nanoparticles are versatile carriers used for delivering therapeutic payloads and diagnostic agents simultaneously (Zhang et al., 2023a; Thangam et al., 2021). The integration of a conductive polymer, polypyrrole (PPy) and mesoporous iron-based metal–organic frameworks (MOF) allowed hybrid photothermal-chemotherapy with delivery (Zhu et al., 2016). Nanodimensioned hydrogels (nanogels) also may be fashioned as soft, multifunctional and biologically responsive drug carriers (Ali et al., 2022). The polymer and payload may be dissolved in a suitable organic solvent (e.g., antigens, drugs, nucleic acids) to form a homogeneous solution. The payload may become encapsulated within the polymer matrix or adsorbed onto its surface. Generally, an emulsion is created by adding the polymer-payload solution to an aqueous phase containing processing aids, such as surfactants or sizing chemicals. Methods like oil-in-water (o/w) or water-in-oil-in-water (w/o/w) emulsification allow for controlled evaporation, by diffusion or coacervation, of the organic solvent that leads to the formation of nanoparticles. Finally, purification removes unencapsulated payload or residual solvents through techniques like ultracentrifugation, dialysis, or filtration (Wibowo et al., 2021). For example, hydrogel (CAHG) is prepared by *in situ* crosslinking of L-arginine (L-Arg)-coupled chitosan and glucose oxidase (GOx)-modified hyaluronic acid based on Schiff-base reaction. The system can mediate a continuous release of hydrogen peroxide (H_2_O_2_) and NO by the cascaded consumption of glucose and L-Arg in the presence of hyperglycemia environment. CAHG hydrogel have excellent biocompatibility and glucose-responsive NO release characteristic can serve as a highly efficient therapeutic strategy for diabetic wound treatment (Zhou et al., 2023).

Gold nanoparticles (AuNPs) have unique tunable optical properties, relative ease of functionalization, and inherent biocompatibility that make them useful for both imaging (due to their strong absorbance, light scattering, and surface plasmon resonance (Jana et al., 2016)) and as carriers for vaccine components (Giljohann et al., 2010). A wide variety of shapes, including spheres, rods, ellipses, pyramids and intricate structures including core-shell and encapsulated, are commonly synthesized by the reduction of gold salts, such as chloroauric acid (HAuCl_4_), using a reducing agent such as sodium citrate, sodium borohydride, or ascorbic acid or by nanolithography (Tan et al., 2005). The size and shape of AuNPs determine their optical properties and can be controlled by adjusting factors such as the concentration of gold salts, the type and concentration of reducing agent, and the reaction temperature. Invariably, particles must be stabilized to prevent aggregation and so are often coated with stabilizing agents such as citrate, thiolated ligands, or polymers. Ligands and polymers offer a path to functionalization with molecules that have specific binding properties, such as antibodies, peptides, or aptamers. This allows for the targeted delivery of vaccines and/or diagnostic agents (Paciotti et al., 2006).

Carbon nanomaterials including single walled carbon nanotubes (SWCNTs), multi-walled carbon nanotubes (MWCNTs), graphene, graphene oxide, and carbon dots have been explored for their ability to transport vaccine components and stimulate immune responses (Ahmadi et al., 2023; Patrick et al., 2023). Amine functionalized SWCNTs [poly (di-allyl-dimethyl-ammonium) chloride and hexamethylene-diamine] allowed for electrostatic conjugation with, for example, extracellular signal-regulated kinase (ERK) siRNA to suppress expression of the ERK target proteins in primary cardiomyocytes (Krajcik et al., 2008). Additionally, these have found favor in thermo-excitable drug release, hybrid photothermal therapy (PTT) (Sobhani et al., 2017; Behnam et al., 2018) and/or photodynamic therapy (PDT) (Wang et al., 2014; Sundaram and Abrahamse, 2020). These materials offer unique properties, including biocompatibility, ease of functionalization, and excellent optical and electronic properties. CNTs may be synthesized by chemical vapor deposition (CVD) wherein a carbon-containing gas, like methane, is decomposed on a catalyst substrate (e.g., iron or nickel) to produce a CNT forest. CNTs may also be synthesized by high-temperature techniques like arc discharge or laser ablation by vaporizing carbon rod electrodes. Graphene is commonly produced by chemical exfoliation of graphite using chemical exfoliation methods, such as the Hummers’ method, which involves oxidizing graphite and then reducing the resulting graphite oxide. Like CNTs, graphite can be produced by CVD wherein single layers of carbon atoms are grown on metallic substrates. Carbon dots may be prepared by hydrothermal/solvothermal methods that involve heating carbon-containing precursors (e.g., citric acid) in the presence of solvents and catalysts under high-pressure conditions. Another path to CDs is via microwave irradiation of organic precursors. Despite the challenging syntheses, purity concerns, and potential toxicities, carbon nanomaterials continue to be pursued as possible platforms for cancer vaccine theragnostics (Asil et al., 2023; Cao et al., 2019; Yin et al., 2022).

Silica nanoparticles can be readily chemically modified, functionalized, and derivatized and so engineered to carry antigens, adjuvants, or imaging agents for cancer diagnostics (Ali et al., 2022; Şen Karaman et al., 2018). Generally regarded as biocompatible, silica nanoparticles may be controllably prepared by the Stöber method that involves the hydrolysis and condensation of silane precursors (e.g., tetraethyl orthosilicate) in the presence of ammonia and water. This produces spherical nanoparticles particles. Silica nanoparticles can be synthesized through a sol-gel process by controlling the hydrolysis and condensation reactions of silane precursors. This method allows for the incorporation of various functional groups during synthesis. A water-in-oil microemulsion method exploits the hydrolysis of silane precursors to occur within nanoscale water droplets. Similar to the microemulsion method, the reverse micelle method allows for synthesis of silica nanoparticles within reverse micelles in an organic solvent. Finally, an emulsion polymerization method, with initiation within the oil droplets, allows the synthesis of silica nanoparticles encapsulated in polymer shells (García-Uriostegui et al., 2022).

Dendrimers are highly branched, organic, polymeric nanoparticles with well defined, tunable, controllable size and structure with capability for convenient surface functionalization (Wang et al., 2022; Lyu et al., 2020). Dendrimers have been used to encapsulate (formulation) or physicochemically conjugate (conjugation) antigens and adjuvants for vaccine delivery (Lyu et al., 2020; Heegaard et al., 2010). When a central core molecule is reacted with a multifunctional monomer (usually containing two or more reactive groups) this forms the first generation of dendrimer. Subsequent generations are created by adding more multifunctional monomers to the existing branches, resulting in a highly branched structure in what is called divergent synthesis. Dendrimer surfaces can be functionalized with various molecules, including antigens, antibodies, or imaging agents, to tailor their properties for vaccine delivery, diagnostics and possibly theragnostics (Abbasi et al., 2014).

Quantum dots (QDs) are semiconductor nanoparticles with unique optical properties that emit fluorescent light (Ekimov, 1981; Rossetti and Brus, 1982; Murray et al., 1993) and can be used for imaging and tracking purposes including imaging, diagnostics, and drug delivery in cancer vaccine development (McHugh et al., 2019). Core-shell QDs possess a core made of a semiconductor material like cadmium selenide (CdSe), cadmium telluride (CdTe), or indium arsenide (InAs), while the shell is usually a wider bandgap semiconductor like zinc sulfide (ZnS). This structure enhances the photostability and fluorescence quantum yield of the QDs. To control size and surface properties, QDs are coated with organic ligands, such as trioctylphosphine oxide (TOPO) or oleic acid. These ligands stabilize the QDs and can be replaced with hydrophilic ligands for aqueous applications. For cancer vaccine diagnostic applications, QDs are often transferred from nonpolar organic solvents to aqueous solutions by exchanging the hydrophobic ligands with hydrophilic ones, like mercaptosuccinic acid or PEG. Smaller QDs emit at shorter wavelengths (blue), while larger QDs emit at longer wavelengths (red). QDs can be functionalized with peptides, antibodies, or aptamers to enable specific targeting of cells or with antigens for diagnostic or therapeutic purposes. If the QDs are intended for vaccine delivery or theranostic applications, they can be further functionalized with vaccine antigens, therapeutic agents, or imaging molecules to create multifunctional QD-based platforms. The potential toxicity of QDs is a relevant consideration (Abdellatif et al., 2022; Le and Kim, 2023).

Virus-like nanoparticles (VLNPs) are self-assembling nanoparticles that are structural mimics of viruses but lack the viral genetic material, thus making them a safe and effective platform for vaccine development (Perotti and Perez, 2019). VLNPs can be engineered to display cancer antigens and stimulate immune responses without causing disease (Nooraei et al., 2021; Arevalo et al., 2016). The gene encoding the viral structural proteins responsible for forming the VLNP is cloned into an expression vector and the chosen expression system transfected with the recombinant expression vector. Protein expression is induced under controlled conditions. The viral structural proteins assemble into VLNPs within the host cells. A benefit of VLNPs is that they can be functionalized with vaccine antigens or epitopes by genetically fusing them to the VLP structural proteins. This allows for the presentation of antigens in their native conformation, enhancing the immune response. VLNPs can be readily labeled with fluorescent dyes or conjugated to other nanoparticles, such as quantum dots, to enable tracking and imaging *in vivo*. In some instances, the imaging beacon can be encapsulated within the VLPs. Similarly, targeting ligands such as antibodies, antibody fragments, or peptides can be attached to the VLNPs to enhance their specificity for receptors and for specific interactions with targeted cells or tissues (Tornesello et al., 2022; Caldeira et al., 2020). VLNPs hold singular promise as an asset in the arsenal in the development of cancer vaccines (Mohsen and Bachmann, 2022).

Iron oxide nanoparticles (IONPs) (Fe_3_O_4_ or γ-Fe_2_O_3_), because they are commercially available, easily synthesized, bio-benign (biocompatibility), and readily capped and functionalized are often used in targeted drug delivery to carry antigens or adjuvants (Chung et al., 2021; Naletova et al., 2023). However, when their magnetic properties are also exploited, such superparamagnetic iron oxide nanoparticles (SPIONs) serve as contrast agents in MRI for cancer diagnosis, specifically used in vaccines due to their magnetic properties for targeted delivery and their imaging capabilities for diagnostics (Wu and Huang, 2017). However, other magnetic nanoparticles, such as manganese oxide (MnO) and cobalt ferrite (CoFe_2_O_4_) may similarly be used. IONPs may be prepared by a co-precipitation method wherein iron salts (e.g., FeCl_3_ and FeCl_2_ or iron sulfate) are mixed with a base (e.g., NaOH or NH_4_OH) under controlled conditions, resulting in the precipitation of iron oxide nanoparticles. A thermal decomposition route involves heating iron precursors, typically organometallic iron compounds, in the presence of surfactants or stabilizers as an approach to monodisperse IONPs with controlled sizes and shapes (Ajinkya et al., 2020). There is also a hydrothermal/solvothermal process that involves high-temperature and high-pressure reactions within a closed vessel, resulting in MNPs with controlled size and morphology. A surfactant stabilized water-in-oil microemulsion or reverse micelles method in which iron precursors are added to an aqueous phase surrounded by an oil phase containing surfactants produces spherical IONPs with increased monodispersity (Salvador et al., 2021). This method allows for better control over particle size, shape, and distribution. Following synthesis, MNPs are typically coated and/or functionalized to improve their stability (reduce reactivity) and impart biocompatibility (Janko et al., 2019; Halder et al., 2022). Accordingly, IONPs are core-shell particles with a shell that is chemically functionalizable with molecules like citrate, dextran, PEG, or polyethylenimine (PEI) that enhance their suitability for biological applications. MNPs benefit from purification methods that employ magnetic separation (Zhao et al., 2020; Lin et al., 2020).

Polymeric micelles are nanoscale aggregates of amphiphilic block copolymers that can encapsulate hydrophobic drugs or imaging agents, enhance antigen stability, and serve as delivery vehicles for both therapeutic and diagnostic agents (Figueiras et al., 2022; Ghezzi et al., 2021). These are biocompatible and biodegradable amphiphilic polymers that can self-assemble into nano-structured micelles (Patra et al., 2018). Prepared from block co-polymers comprising PEG, PLGA, poly (caprolactone) (PCL), polyethylenimine (PEI), poly (2-oxazoline)s and/or poly (N-vinyl pyrrolidone) (PVP), these polymers may be molecularly engineered to achieve tailored properties (Lee et al., 2019). The choice of polymer can influence the micelle’s properties, including stability, drug loading capacity, and drug release profile (Haider et al., 2020). This modification enables the polymer to form a hydrophobic core within the micelle, capable of encapsulating hydrophobic vaccine components or drugs. Being polymers, these nanoparticles are readily functionalized (have an abundance of reactive chemical functional groups) via chemical covalent conjugation with targeting ligands, antibodies, or diagnostic imaging agents, enabling specific interactions with cells or tissues. An endearing feature of polymeric micelles is the relative ease with which stimuli-responsive properties may be conferred (Wells et al., 2019) allowing them to alter payload delivery in response to changes in pH, temperature, or enzyme activity for the controlled release of drug or diagnostic signal activation (Biswas et al., 2016; Gerardos et al., 2023).

Protein-based nanoparticles such as albumin nanoparticles or virus-derived nanoparticles, can be used to deliver antigens or drugs in cancer vaccines. Compatible proteins such as bovine serum albumin, ovalbumin, or viral coat proteins (e.g., capsid proteins) may form the building blocks for these nanoparticles. They may be formed via self-assembly, exploiting the inherent properties of proteins to aggregate. Alternatively, protein’s amino acid sequence may be modified through genetic engineering techniques to introduce self-assembly motifs, crosslinking sites, or fusion tags that facilitate nanoparticle formation and conjugation. Protein molecules can self-aggregate into nanoparticles due to hydrophobic interactions, electrostatic forces, or promoted by covalent cross-linking using agents such as glutaraldehyde or EDC (1-ethyl-3-(3-dimethylaminopropyl)carbodiimide), promoting nanoparticle formation, stability and resistance to degradation. This method allows for precise control over the size and stability of the nanoparticles particularly under controlled pH, ionic strength (salt concentration) and temperature conditions. Another approach, nanoprecipitation, is a technique where a protein solution is mixed with a non-solvent or a precipitating agent to induce nanoparticle formation through the controlled phase separation of the protein. Multifunctional protein nanoparticles that chelate cobalt ions, targets and partitions into mitochondria, induces reactive oxygen species (ROS) production and reduces the mitochondrial membrane potential. The resulting *in vivo* antitumor activity synergistically suppresses tumors and prolongs survival when covalently conjugated with paclitaxel (Zhu et al., 2019).

Polymer composites and microfabricated carriers are engineered, implantable-based composite systems for anti-cancer drug delivery and represent a general approach to enable cancer theragnostics using RNA therapy. A wide range of natural and synthetic polymers may be fashioned with anti-cancer drugs into composites that serve to enhance delivery or efficiency (Hazra et al., 2021; Bakhshi et al., 2024; Mohebian et al., 2021). Such engineered systems include 3-D printed drug eluting composites (Ullah et al., 2023; Bisht et al., 2024; Wang et al., 2020; Gangrade and Mandal, 2020) but may include e-jet printed (Yang et al., 2020), microfluidics prepared nano-microspheres (Zhang et al., 2023b), coacervates (Kim et al., 2022) and the like of drug-biomaterial composites (Mondal et al., 2023). Such systems hold great potential in closed-loop theragnostics, if they could be rendered active in response to potentiated levels of therapeutically modulated biomarkers. This includes microfabricated systems with multiple drug reservoirs and with multiple response profiles, each engineered to be actuated in response to a particular biomarker level (Koch et al., 2016; Pirmoradi et al., 2013).

### Characterization and regulatory considerations in the use of nanoparticles

In the context of cancer vaccine development, the synthesis or preparation of NPs is pursued to address specific performance requirements, is done with rigorous application of statistical experimental design techniques (e.g., Taguchi), and is accompanied by vigorous characterization (Aikins et al., 2020). NPs are generally characterized for i) particle size and distribution using techniques such as dynamic light scattering (DLS) or nanoparticle tracking analysis (NTA), ii) surface charge (Zeta Potential) using electrophoretic mobility techniques, iii) morphology using transmission electron microscopy (TEM) or scanning electron microscopy (SEM) or atomic force microscopy to establish the shape(s) of the nanoparticles, iv) structural analysis using techniques like circular dichroism (CD) or nuclear magnetic resonance (NMR) spectroscopy to analyze the secondary and tertiary structure of proteins in or on the nanoparticles, v) payload encapsulation efficiency using techniques such as gel electrophoresis or UV-visible spectroscopy, vi) Stability using the influence of temperature, pH, and ionic strength to ensure that NPs remain intact during storage and administration, vii) payload release kinetics and modeling under simulated physiological conditions to determine how effectively the NPs deliver their cargo, and viii) *in vitro* and *in vivo* studies to assess cytotoxicity and their ability to transfect target cells. *In vivo* studies in animal models provide insights into biodistribution and immunogenicity. A key feature of theragnostic NPs is their ability to convey diagnostic information via chemically conjugated imaging probes or biomarkers without compromising vaccine efficacy. Process and product scalability are also crucial to ensure that the synthesis process can produce NPs in quantities suitable for pre-clinical characterization, clinical trials, and large-scale vaccine production. Moreover, the distribution of RNA among and within the NPs must result in a minimum number of NPs being unoccupied. Finally, all NP-based cancer vaccines must meet regulatory compliance requirements for safety and efficacy in vaccine development and diagnostics. NPs should be non-cytotoxic and maintain their biocompatibility and safety profiles for clinical applications. The choice of nanoparticle depends on the specific requirements of the cancer vaccine, including the type of antigen, desired release profile, and the diagnostic or imaging modality being used. However, a central dogma of the cancer vaccine theragnostic is the stimuli responsive activation of the payload release in response to some activating signal. These signals should ideally originate from within the targeted cell or tissue and potentiate the release of the RNA payload. However, such activation may also originate from outside of the cells or tissues or even outside of the body when in response to guided diagnostic information from those cells and tissues enabled by the cancer vaccine theragnostic platform. Researchers continue to explore and develop new nanoparticle-based platforms for cancer vaccine theragnostics to improve the effectiveness of cancer diagnosis and treatment.

## Methods of theragnostic activation

Theragnostics platforms require an activation step wherein a signal from, or associated with the target, is returned to the delivery platform indicating that it’s suitable to release the payload. This feedback control step we call theragnostic activation. This unique stimuli-responsive characteristic distinguishes a theragnostic platform from a delivery or diagnostic or combination delivery-diagnostic platform. Such systems are subject to the theories and engineering principles of control systems and must comprise the four components of stimulus, sensor or sensory receptor, a control or set point center, and an effector, a concept introduced and expanded in our earlier work (Wilson and Guiseppi-Elie, 2013). Theragnostic activation may be exogenous, arising from outside the body, or endogenous, arising from within the body. Exogenous stimuli include light, magnetic fields, temperature, electric fields, and mechanical (e.g., cavitation via ultrasound) (Pham et al., 2020). Endogenous stimuli include physiologically or pathophysiologically derived physio-chemical stimuli such as chemical potential gradients (e.g., pH and redox gradients) and enzyme or hormone activities. Following are illustrative examples of platform activation using light, redox potential, electric fields, and mechanical forces (ultrasound).

### Photo-activatable platforms

Photo-activatable platforms rely upon the application of light (photons) to realize activation of the release of the drug in a stimuli responsive manner (Tao et al., 2020; Rapp and DeForest, 2021). Pan et al. (2021) have reviewed clinical and experimental applications in cancer treatment of photosensitive drug release systems, including nanocarriers such as liposomes, micelles, polymeric nanoparticles, and hydrogels (Pan et al., 2021). Wan et al. (2023) have reported a photoactivatable nanoagonist platform that confers near-infrared (NIR) light-induced cytotoxicity and immunogenic cell death concomitant with NIR light-triggered agonist release for immunotherapy (Wan et al., 2023). Photodegradable hydrogels possessing the photo-labile orhonitrobenzyl moiety within their polymeric backbone have been used to release siRNA. Upon UV irradiation, the siRNA is released to achieve, in this case, knock down expression of model proteins (e.g., green fluorescent protein, luciferase) in cultured HeLa cells (Huynh et al., 2016) and to direct osteogenesis of human mesenchymal stem cells (Huynh et al., 2017). Researchers at the Paul Scherrer Institute have made a film that could give a decisive boost to developing a new type of drug. They made the advance in the field of photopharmacology, where substances can be activated or deactivated with the help of light (Wranik et al., 2023). However, few studies focus on RNA therapeutics and cancer vaccination. A creative combination of gene therapy and photothermal therapy was achieved by using polyetherimide-modified single-wall carbon nanotube (PEI-SWNT) and Hsp70B′-promoter-driven RNAi vector (pHSP-shT). The (PEI-SWNT)/pHSP-shT was responsive to NIR heating that triggered gene knockdown targeting human telomerase reverse transcriptase through RNAi in MCF-7 breast cancer cells (Xueling Ren et al., 2017). Biomimetic nanogels developed by Luo and Shi can be activated by NIR to co-deliver temozolomide and indocyanine green to deep tumor, so that orthotopic glioblastoma can be effectively inhibited (Zhang et al., 2022a). While light-based activation techniques offer excellent spatial and temporal control, versatility via wavelength and intensity selection, and minimum side effects, continued challenges include depth of penetration, costly systems, and safety concerns regarding photo-activatable chemistries and materials.

### Redox-activatable platforms

Redox-activatable platform depends upon a change in redox state to serve as a triggering signal to effect a change in the delivery platform, resulting in the release of the drug. Such redox state differences exist within vacuoles, inclusion bodies, within solid tumors, etc. As an example, novel redox-responsive amphiphilic nanoparticles of the disulfide-lenalidomide-methoxy PEG were generated for the non-invasive co-delivery of a conventional chemotherapy drug methotrexate and an anti-angiogenic drug lenalidomide to the brain through the lymphatic vasculature, which may serve as the beginning of the new strategy to treatment of primary central nervous system lymphoma (Liu et al., 2023). ROS, which often elevated in the cancer and stress circumstances, may also serve as effectors in the feedback control that establishes the theragnostic. Self-assembled SPM-based metal-immunopeptide nanocomplexes (APP-Fe NCs; APP is anti-programmed death ligand-1 peptide) with pH- and ROS-responsive release boosted ferroptotic immunotherapy of lymphoma and can be applied as a sensitive theragnostic platform (Nie et al., 2023). ROS-responsive and Raman-traceable hydrogel based on a degradable conjugated polymer poly (deca-4,6-diynedioic acid) (PDDA) has been developed to integrate Raman imaging-guided photodynamic and immune therapy for postsurgical cancer treatment (Zhang et al., 2022b).

### Electro-activatable platforms

Electro-activatable delivery platforms rely upon the use of exogenous electric fields to realize activation of the release of the RNA therapeutic (Olvera and Monaghan, 2021). Such electric fields may be produced by implantable electrodes, such as are found in medical devices or topically affixed electrodes, such as are found in wearable electronics. High-intensity exogenous electric fields may be used to directly alter the permeability of cellular membranes, in a form of *in vivo* electroporation (Muramatsu et al., 1998; Matsuda and Cepko, 2004). Accordingly, such fields may enhance transmembrane permeability of uniquely conjugated RNA. Electric fields may also alter the *in vivo* release characteristics of RNA-loaded nanomaterials by altering the physico-chemical properties of the nanomaterials or by affecting interactions between the RNA and the carrier nanomaterial. Electric fields can establish electrophoretic forces that move charged molecules, such as RNA, away from the nanocarriers, particularly if they are immobilized by secondary forces. Nanomaterials, particularly those made of electro-responsive hydrogels, may undergo redox or surface conformational changes that induces the release of the RNA payload. Such changes can alter pore size or the structural integrity of the nanomaterial, affecting how the RNA is released over time. Electric fields may also alter the ionic environment around the nanomaterials that influences the electrostatic interactions between chemisorbed RNA and the nanocarrier. This, in turn, might promote or hinder the release of RNA. In some cases, electric fields can induce local electrochemical reactions that alter the surface properties of the nanomaterial, leading to changes in how RNA is bound or released. This area of research is particularly promising for controlled drug delivery, where electric fields could be used to fine-tune the release profiles of RNA. Finally, electric fields may modulate the reactivity of field-responsive reactions that govern the uptake and expression of mRNA (Fried and Boxer, 2017).

### Mechano-activatable platforms

Mechano-activatable platforms employ exogenous mechanical forces, typically ultrasound, to realize activation of the release of drugs (Ma et al., 2022; Shi et al., 2024). Ultrasound, because it is noninvasive, highly focused, and affords strong tissue penetration, is a good activation mechanism for RNA theragnostics (Liu et al., 2024; Bravo-Vázquez et al., 2023). The cavitation energy of ultrasound exerts mechanical forces on nanomaterial-loaded RNA. Such mechanical forces may originate from ultrasound devices, such as are found in diagnostic imaging systems (Pan et al., 2024). Wang et al. (2016) developed and applied ultrasound-responsive microbubbles that were loaded with siRNA targeting the X-linked inhibitor of apoptosis protein (XIAP) to serve as a hybrid therapeutic-diagnostic in the treatment of cancer. Real-time monitoring of siRNA drug efficacy and improved penetration was achieved using ultrasound (Wang et al., 2016). PEGylated PLGA nanoparticles loaded with microRNA (miR-122) were delivered *in vivo* to human colon cancer-bearing mice (xenografts) under ultrasound guidance, achieving an enhanced therapeutic effect (Wang et al., 2015). In the work of Yang et al. (2015), PLGA nanobubbles containing DOX and P-glycoprotein short hairpin RNA (shRNA) were shown to effectively combat tumor drug resistance and to simultaneously enable real-time guidance during treatment using ultrasound (Yang et al., 2015). Ultrasound, via microbubble cavitation, is a useful adjunct for delivery of therapeutic RNA loaded nanocarriers for cancer theragnostics.

## Theragnostics using RNA therapeutic agents

Small interfering RNAs (siRNA) and antisense oligonucleotides (ASO) opens a new mechanism for the regulation of post-transcriptional processes. In 2018, siRNA demonstrated its therapeutic efficacy and the first siRNA drug, *patisiran*, was approved by the USFDA (Friedrich and Aigner, 2022). The major challenges in RNA therapeutics are effective delivery, cellular uptake and endosomal escape, stability, and off-targets (Paul et al., 2022). To address these limitations new chemical modifications of siRNA and ASOs are under investigation. *N*-alkyl phosphoramidates (mesyl and buzyl) represent new chemical modification of oligonucleotides, which may be applied for the validation of new therapy targets and future application in the therapeutics ASOs for treatment of orphan diseases and cancer (Miroshnichenko et al., 2019; Hammond et al., 2021). Spinal muscular atrophy (SMA) is a leading genetic cause of infant mortality, primarily due to motor neuron degeneration and progressive muscle weakness. In 2016 the USFDA approved *nusinersen* (Spinraza), a drug based on chemically modified ASO for SMA treatment. Spinraza works by increasing the function of a gene that is defective in people with SMA and is given as an injection into the fluid surrounding the spinal cord. This clinically approved antisense oligonucleotide drug is an 18-mer oligo-2′-O-(2-methoxyethyl) (2′-MOE) ribonucleotide with a phosphorothioate backbone. Phosphorothioate groups (PS) improve cellular uptake, biodistribution and pharmacokinetic properties of the oligonucleotides. However, there are several adverse effects of PS modifications that have since been documented, most notably, *in vivo* toxicity, particularly liver damage and complement activation (Webb et al., 2001). The concerns over clinical safety of PS oligonucleotides had resulted in the repeated refusals of the European Medicines Agency (EMA) to approve mipomersen (Kynamro), an FDA-approved ASO inhibitor drug targeted to apolipoprotein B-100 (apoB-100) mRNA that is administered via subcutaneous injection to treat homozygous familial hypercholesterolemia (Wong and Goldberg, 2014). Mipomersen causes selective degradation of the apoB-100 mRNA and consequent inhibition of protein translation. There are documented failures of several PS-modified drug candidates to make it through different phases of clinical trials. It was demonstrated that new chemical modifications mesyl (methanesulfonyl) and busyl (1-butanesulfonyl) phosphoramidate groups can be an efficient alternative to phosphorothioate (PS) group in antisense oligonucleotides. The University of Oxford, UK study of mesyl and busyl modifications in splice-switching ASO, which leads to increased stability of antisense oligonucleotide duplexes with RNA and completely abolishes Rnase H activation, was essential for splice-switching activity in SMA patient derived fibroblasts and *in vivo* in a neonatal mouse model of SMA. This study, with detailed analysis of the application of the new chemically modified ASOs for SMA treatment, additionally validated SMN2 mRNA as a therapeutic target (Hammond et al., 2021). Additionally, Ionis demonstrated that replacing two or more PS near the 5′-side of the gap in ASO gapmer with mesyl groups reduced cytotoxicity and hepatotoxicity *in vivo*. Replacing PS with mesyl near the 5′-side of the gap was effective at the reduction of the pro-inflammatory profiles, suggesting that ASO interactions with proteins of the immune system may be affected by the chemical modification and that replacing PS with mesyl groups can be a general chemical strategy to mitigate these interactions and reduce pro-inflammatory effects (Anderson et al., 2021). These data demonstrated that chemical modifications of ASO or siRNA influences on its stability and other physicochemical properties, which can be used for the development theragnostics platform. The successful application of siRNA for cancer theragnostics also requires the development and application of suitable, safe and effective delivery systems, like NPs, which are widely applied in therapeutics, diagnostic imaging agents, *in vitro* diagnostics, and medical devices (Figure 1). Several imaging modalities such as MRI, ultrasound, optical imaging, including bioluminescence and fluorescence imaging, SPECT, and PET are being integrated into siRNA theragnostic nanomedicine by incorporating the relevant imaging reporter into the NP (Massoud and Gambhir, 2003). Contrast agents, such as gadolinium and manganese, that modify relaxation time of the water signal can be used to detect the delivery of theranostic nanoplatforms delivering siRNA (Li et al., 2008). Synthetic polyamines like PEI have been widely applied in siRNA delivery. Incorporation of PEI into a prostate specific membrane antigen (PSMA)-targeted nanoplatform with a poly-L-lysine backbone, carrying multimodality imaging reporters together with siRNA or cDNA and a prodrug enzyme for cancer theragnostic imaging, has been achieved (Chen et al., 2012; Chen et al., 2017a). The prodrug enzyme modified a nontoxic prodrug 5-fluorocytosine to the chemotherapy agent, 5-fluorouracil, which can be detected by 19F MRS while the nanoparticle platform carried siRNA for the downregulation of the oncogene in the cancer cells.

Another illustrated approach is the development and application of redox-activatable theragnostic platform based on the ROS-biodegradable siRNA nanocarrier, which is opened within the cancer cells (Juan et al., 2021). Also, there are several works for the development of targeted liposomes, such as a PEGylated cationic liposome, for the therapeutic delivery of siRNA to cancer cells. For example, PEGylated cationic liposome with an aptamer AS1411 with the selective binding to nucleolin overexpressed on the surface of cancer cells. This nucleolin-targeting liposome was used to deliver anti-BRAF siRNA to treat an A375 melanoma model. Imaging of Cy5.5 labeled siRNA demonstrated tumor specific delivery despite some renal accumulation, and tumor growth inhibition (Li et al., 2014). Novoselova et al. (2023) investigated nanocarriers with DARPin polypeptide scaffold targeting HER2 receptors that are overexpressed in 20%–30% of breast cancers. Select nanocarriers had a rather high sensitivity to low intensity focused ultrasound (LIFU) and primarily interacts with mitochondria within cancer cells (Novoselova et al., 2023). The targeted delivery of therapeutic agents followed by externally applied low intensity focused ultrasound (LIFU) represents a type of “theragnostic” wherein the stimulatory activation is gated via human intervention (Liu et al., 2019).

An approach which comes quite close to a dedicated theragnostic platform is the development of a ROS-responsive siRNAs via chemical modification of therapeutic oligonucleotides with 4-boronobenzyl (4BB) groups. This resulted in an antisense theragnostic for ROS-rich cancer cells. The ROS response produced efficient cleavage of the 5′-4BB-siRNA resulting in activation of siRNA-based prodrugs in cell-free settings, within an immortalized ovarian endometroid adenocarcinoma tumor cell line and murine hepatoma cell line, which are known to have high intracellular ROS levels, as well as in the liver of adult mice after partial hepatectomy (conditions of high oxidative stress). The resulting prodrugs had similar pharmacological properties (e.g., safety, pharmacokinetics, pharmacodynamics, etc.) to classical siRNAs, which promote their preclinical and clinical development (Rühle et al., 2022). In yet another example, Shi and co-workers have reported a 3l-NM@siRNA system, which is responsive to endogenous ROS present in tumors with the effective at-site siRNA release resulting from tumoral ROS-triggered sequential destabilization (Zheng et al., 2019). The platform-based integration of targeting with ROS-activation to produce a theragnostic.

### Examples of theragnostics for metastatic castration resistant prostate cancer

Prostate cancer (PC) is the second most common cancer and sixth leading cause of cancer related death among men in the world with 10%–20% patients eventually developing castration resistant prostate cancer (CRPC) and with 84% of patients having metastases at the time of their cancer diagnosis (Andriole et al., 2019). For this type of cancer there is a unique marker—prostate specific membrane antigen (PSMA), which is highly and selectively expressed on prostate cancer cells. The theragnostic concept of PC is based on PSMA overexpression in the cancer cells, which allows the use of PSMA ligands for systemic therapy in patients with metastatic PC. ProstaScint^®^ was an ^111^In-labeled anti-PSMA monoclonal antibody, which was the first PSMA targeted imaging agent in a SPECT approved by the FDA in 1996. Unfortunately, the antibodies had low tumor penetration and high background and thus represented limited diagnostic potential (delayed target recognition, low tumor-to-background ratios) (Bander, 2006). Small molecule PSMA inhibitors found application as PET agents and demonstrated superior detection rates and accuracy compared to anatomical imaging. Radiolabeled ^68^Ga-PSMA-11 and ^18^F-DCFPyL were approved by the FDA in 2020 and 2021 for PC patients at high risk for pelvic nodal metastases and biochemical recurrent prostate cancer (Eder et al., 2012). Next-generation of PSMA theragnostic ligands, like ^177^Lu-RPS-063, had the albumin-binding moiety and the PSMA-binding moiety, which allowed the relative affinities for PSMA and serum albumin to be fine-tuned (Kelly et al., 2018). Development of new PSMA targeting ligands occurs in parallel with the development of novel chelators that complexes for application in the imaging and therapy. Radioisotopes of copper are re-emerging for theranostic application in prostate cancer. For example, ^64^Cu-PSMA-ALB-89 with albumin binding groups accumulates in both PC3-PIP xenografts and kidneys (Umbricht et al., 2018), while ^64^Cu-RPS-085, a sarcophagine chelator, clears more rapidly from kidneys (Kelly et al., 2020). A new radiometal β-emitters for PSMA-targeted radioligand therapy, such as ^43/44/47^Sc (Vaughn et al., 2021), ^67^Cu (Kelly et al., 2020; McInnes et al., 2021) and ^161^Tb (Müller et al., 2019), and α-emitters, such as ^149^Tb (Umbricht et al., 2019), ^212^Pb (Stenberg et al., 2021), ^227^Th (Hammer et al., 2020), and ^225^Ac are under development and become more widely available, because of their higher *in vivo* stability.

So far, PSMA-targeted liposome-like lipid nanoparticles with the possibility of the ^111^In and Lu chelation for simultaneous SPECT/CT imaging and PDT were developed (Cheng et al., 2021b). Lipid nanoparticles enable the encapsulation of both hydrophilic and lipophilic drug molecules, which may be used for the therapy. Nanoparticles conjugated with PSMA enabled the specific accumulation in PSMA + PC3-Pip tumors and reached the peak at the 8 h time point with 2-fold higher uptake in the tumors. Recombinant single-chain antibodies are also highly potent targeting ligands, but may undergo rapid clearance (Muñoz-López et al., 2022).

Combination of the prodrug enzyme therapy strategy with siRNA may improve cancer-selective therapy. Bhujwalla and co-workers demonstrated a PSMA-targeted nanoplex platform for PCa theragnostics by delivering a prodrug enzyme with siRNA (Chen et al., 2012; Bhujwalla et al., 2018). The nanoplexes were designed with a NIR fluorescent probe Cy5.5, a prodrug-activating enzyme, bacterial cytosine deaminase (bCD), and radiometal ^111^In chelator for SPECT imaging. Сytosine deaminase bCD converted the nontoxic prodrug 5-fluorocytosine (5-FC) to 5-fluorouracil (5-FU) and siRNA targeted choline kinase can enhance the effect of 5-FU. The authors demonstrated the efficient PSMA-targeted tumor accumulation, but unfortunately there was the simultaneous high uptake of the nanoplex in the liver and kidneys. There has been several noteworthy updates since the early work of Bhujwalla (Friedlaender, 2023; Kaewput and Vinjamuri, 2022). The development of the multimodality imaging reporters together with prodrug enzyme and siRNA may be advantageous in the theragnostic application in metastatic PCa and may also be extended to other cancer subtypes and therapeutic targets.

### Opportunities and challenges

**FIGURE 5:**
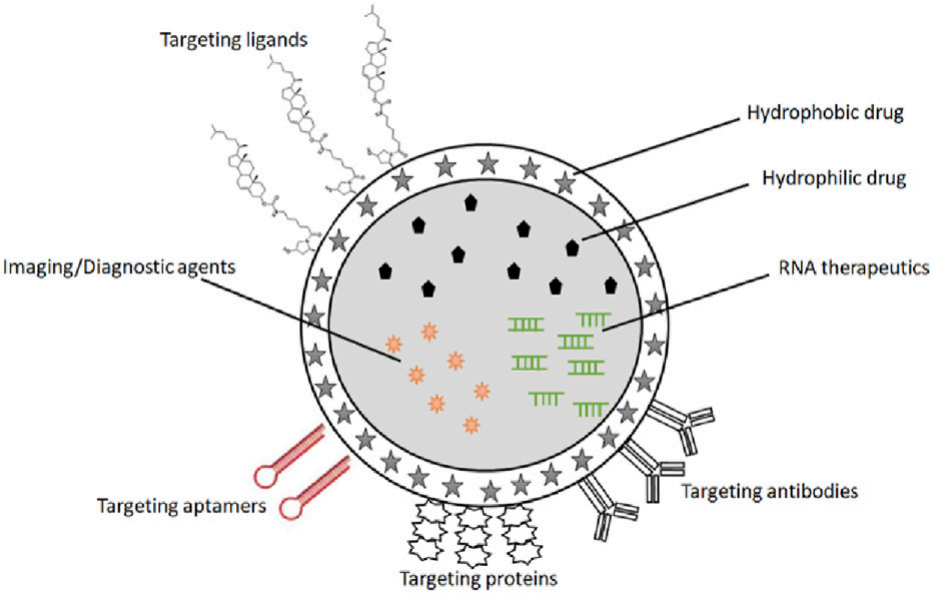
Schematic illustration of a potential theragnostic nano-delivery platform for cancer vaccines and RNA therapeutic drugs. The theragnostic platform shows: different targeting ligands and molecules; drug-loaded nanoparticle core, e.g., lipid nanoparticle with siRNA, ASO or RNA aptamer; imaging reporter or contrast agent to allow diagnostic tracking of the nanoparticles.

Theragnostics offer significant opportunities for the development and personalization of cancer vaccines and RNA therapy drugs (Figure 5). By providing real-time monitoring of treatment response, theragnostics can enable personalized treatment adjustments and improve outcomes for patients.

In addition, cancer theragnostics can help to identify patients who are likely to respond to immunotherapy, enabling a more targeted and effective approach to therapy. However, there are also significant challenges associated with the development and translation of cancer theragnostic vaccines and new RNA therapy into clinical practice. These include the need for more sensitive and specific diagnostic tools, the optimization of imaging and biomarker protocols, and the integration of theragnostics into clinical workflows. In addition, the high cost of some diagnostic tools may limit their accessibility to patients, highlighting the need for cost-effective approaches to theragnostics.

Circulating tumor cells (CTCs) represent an important target in closed-loop theragnostics. CTCs are cancer cells that have shed from the primary tumor, entered the bloodstream and are a crucial source for cancer metastasis (Chen et al., 2017b). Recently thoroughly reviewed (Lawrence et al., 2023; Allen, 2024), their detection, isolation, and analysis have emerged as a promising approach for the diagnosis, prognostication, and therapeutic monitoring of a wide range of cancers (Ahn et al., 2021; Habli et al., 2020), including breast cancer (Jin et al., 2020), lung cancer (MALY et al., 2019), hepatocellular carcinoma and head and neck cancers (Hazra et al., 2020). Process methods to efficiently capture, concentrate and isolate CTCs are being aggressively pursued (Bankó et al., 2019; Descamps et al., 2022; Singh et al., 2021). Among them magnetic capture appears promising with the use of magnetically-activated, nanostructured cellulose nanocrystals (CNCs) or cellulose nanofibers (CNFs) that trap CTCs allowing magnetic isolation and extraction (Li et al., 2021; Hazra et al., 2024). Most technologies are, however, *ex vivo*, necessitating “clinician-in-the-loop” theragnostics (Bankó et al., 2019). *In vivo* technologies that allow closed-loop control of the delivery of associated RNA therapies will be required (Mazidi et al., 2022). Among the many challenges to the use of CTCs in closed-loop theragnostics include low abundance, heterogeneity, and immunological stealth.

### Future directions

The development and translation of theragnostics is a rapidly evolving field, with significant opportunities for further innovation and progress. Some potential future directions for theragnostics in the development and personalization of cancer vaccines and RNA therapy include:

1. Integration of multi-modal imaging and biomarker analysis: The combination of multiple imaging modalities and biomarker analysis can provide a more comprehensive view of the patient’s immune response to the vaccine or RNA therapy, enabling more accurate monitoring of treatment response and personalized treatment adjustments (Roelofsen et al., 2022).
2. Development of novel theragnostic agents: The development of novel theragnostic agents that combine diagnostic and therapeutic capabilities in a single molecule or nanoparticle could further improve the specificity and sensitivity of therapy monitoring (Thangam et al., 2022; Cheng et al., 2021a).
3. Incorporation of artificial intelligence (AI) and machine learning (ML) algorithms: The use of AI and ML algorithms can enable more accurate and efficient analysis of imaging and biomarker data and facilitate the identification of new biomarkers and treatment targets (Koh et al., 2022; Hou et al., 2022; Shreve et al., 2022).
4. Development of cost-effective theragnostic approaches: The development of cost-effective theragnostic approaches, such as the use of widely available imaging modalities or non-invasive biomarker analysis, could improve the accessibility and affordability of cancer vaccine monitoring for patients (Kemp and Kwon, 2021).
5. Personalized vaccine design based on patient-specific immune profiles: The use of patient-specific immune profiling to guide the selection of vaccine antigens could further enhance the efficacy and personalization of cancer vaccines (Zhao et al., 2021b).
6. The fusion of biosensor-derived molecular fingerprinting of biomarkers of cancer (Justyna Frączyk et al., 2018) with actuation under engineered feedback control will enable personalized theragnostics (Muluneh and Issadore, 2014).

## Conclusion

Theragnostics offer significant opportunities for the development and personalization of new therapy approaches and cancer vaccines, including a new focus on achieving targeted levels of immune response to therapy via closed-loop feedback control of the delivered RNA therapeutic. By combining diagnostic and therapeutic capabilities in a single agent, platform or system, theragnostics can enable real-time monitoring of treatment response and automated personalized treatment adjustments. This is achieved through modulating the innate response and by titrating the stimulating factor under endogenous or exogenous adaptive control. However, there are also significant challenges associated with the development and translation of theragnostics into clinical practice, including the need for more sensitive and specific diagnostic tools, the optimization of imaging and biomarker protocols, and the integration of theragnostics into clinical workflows. The molecular engineering of nanoparticles to integrate response to feedback of the modulated immune response continues to be a challenge. Nonetheless, with continued innovation and collaboration across multiple disciplines, cancer vaccine theragnostics hold great promise for improving cancer vaccine efficacy and patient outcomes.

## References

[B1] Abbasi E. Aval S. F. Akbarzadeh A. Milani M. Nasrabadi H. T. Joo S. W. (2014). Dendrimers: synthesis, applications, and properties. Nanoscale Res. Lett. 9 (1), 247. 10.1186/1556-276x-9-247 24994950 PMC4074873

[B2] Abdel-Hakeem M. S. Manne S. Beltra J.-C. Stelekati E. Chen Z. Nzingha K. (2021). Epigenetic scarring of exhausted T cells hinders memory differentiation upon eliminating chronic antigenic stimulation. Nat. Immunol. 22 (8), 1008–1019. 10.1038/s41590-021-00975-5 34312545 PMC8323971

[B3] Abdellatif A. A. H. Younis M. A. Alsharidah M. Al Rugaie O. Tawfeek H. M. (2022). Biomedical applications of quantum dots: overview, challenges, and clinical potential. Int. J. Nanomedicine 17, 1951–1970. 10.2147/ijn.s357980 35530976 PMC9076002

[B4] Adams D. Gonzalez-Duarte A. O’Riordan W. D. Yang C.-C. Ueda M. Kristen A. V. (2018). Patisiran, an RNAi therapeutic, for hereditary transthyretin amyloidosis. N. Engl. J. Med. 379 (1), 11–21. 10.1056/nejmoa1716153 29972753

[B5] Adepu S. Ramakrishna S. (2021). Controlled drug delivery systems: current status and future directions. Molecules 26 (19), 5905. 10.3390/molecules26195905 34641447 PMC8512302

[B6] Ahmadi S. Ebrahimi Warkiani M. Rabiee M. Iravani S. Rabiee N. (2023). Carbon-based nanomaterials against SARS-CoV-2: therapeutic and diagnostic applications. OpenNano 10, 100121. 10.1016/j.onano.2023.100121 PMC9937779 40478147

[B7] Ahn J. C. Teng P. C. Chen P. J. Posadas E. Tseng H. R. Lu S. C. (2021). Detection of circulating tumor cells and their implications as a biomarker for diagnosis, prognostication, and therapeutic monitoring in hepatocellular carcinoma. Hepatology 73 (1), 422–436. 10.1002/hep.31165 32017145 PMC8183673

[B8] Aikins M. E. Xu C. Moon J. J. (2020). Engineered nanoparticles for cancer vaccination and immunotherapy. Acc. Chem. Res. 53 (10), 2094–2105. 10.1021/acs.accounts.0c00456 33017150 PMC7871038

[B9] Ajinkya N. Yu X. Kaithal P. Luo H. Somani P. Ramakrishna S. (2020). Magnetic iron oxide nanoparticle (IONP) synthesis to applications: present and future. Mater. (Basel) 13 (20), 4644. 10.3390/ma13204644 PMC7603130 33080937

[B10] Alhmoud J. F. Woolley J. F. Al Moustafa A.-E. Malki M. I. (2020). DNA damage/repair management in cancers. Cancers 12 (4), 1050. 10.3390/cancers12041050 32340362 PMC7226105

[B11] Ali A. A. Al-Othman A. Al-Sayah M. H. (2022). Multifunctional stimuli-responsive hybrid nanogels for cancer therapy: current status and challenges. J. Control. Release 351, 476–503. 10.1016/j.jconrel.2022.09.033 36170926

[B12] Allen T. A. (2024). The role of circulating tumor cells as a liquid biopsy for cancer: advances, biology, technical challenges, and clinical relevance. Cancers 16 (7), 1377. 10.3390/cancers16071377 38611055 PMC11010957

[B13] Anderson B. A. Freestone G. C. Low A. De-Hoyos C. L. Iii W. J. D. Østergaard M. E. (2021). Towards next generation antisense oligonucleotides: mesylphosphoramidate modification improves therapeutic index and duration of effect of gapmer antisense oligonucleotides. Nucleic Acids Res. 49 (16), 9026–9041. 10.1093/nar/gkab718 34417625 PMC8450106

[B14] Andriole G. L. Kostakoglu L. Chau A. Duan F. Mahmood U. Mankoff D. A. (2019). The impact of positron emission tomography with 18F-fluciclovine on the treatment of biochemical recurrence of prostate cancer: results from the LOCATE trial. J. Urol. 201 (2), 322–331. 10.1016/j.juro.2018.08.050 30179618 PMC7007765

[B15] Angell H. Galon J. (2013). From the immune contexture to the Immunoscore: the role of prognostic and predictive immune markers in cancer. Curr. Opin. Immunol. 25 (2), 261–267. 10.1016/j.coi.2013.03.004 23579076

[B16] Antonarelli G. Corti C. Tarantino P. Ascione L. Cortes J. Romero P. (2021). Therapeutic cancer vaccines revamping: technology advancements and pitfalls. Ann. Oncol. 32 (12), 1537–1551. 10.1016/j.annonc.2021.08.2153 34500046 PMC8420263

[B17] Arevalo M. T. Wong T. M. Ross T. M. (2016). Expression and purification of virus-like particles for vaccination. J. Vis. Exp. 112, 54041. 10.3791/54041 PMC4927761 27286040

[B18] Arnold C. (2022). Theranostics could be big business in precision oncology. Nat. Med. 28 (4), 606–608. 10.1038/s41591-022-01759-6 35440719

[B19] Asil S. M. Guerrero E. D. Bugarini G. Cayme J. De Avila N. Garcia J. (2023). Theranostic applications of multifunctional carbon nanomaterials. View (Beijing) 4 (2), 20220056. 10.1002/viw.20220056 37426287 PMC10328449

[B20] Baden L. R. El Sahly H. M. Essink B. Kotloff K. Frey S. Novak R. (2020). Efficacy and safety of the mRNA-1273 SARS-CoV-2 vaccine. N. Engl. J. Med. 384 (5), 403–416. 10.1056/nejmoa2035389 33378609 PMC7787219

[B21] Bakhshi V. Poursadegh H. Amini-Fazl M. S. Salari D. Javanbakht S. (2024). Synthesis and characterization of bio-nanocomposite hydrogel beads based on magnetic hydroxyapatite and chitosan: a pH-sensitive drug delivery system for potential implantable anticancer platform. Polym. Bull. 81 (8), 7499–7518. 10.1007/s00289-023-05072-1

[B22] Bander N. H. (2006). Technology insight: monoclonal antibody imaging of prostate cancer. Nat. Clin. Pract. Urol. 3 (4), 216–225. 10.1038/ncpuro0452 16607370

[B23] Bankó P. Lee S. Y. Nagygyörgy V. Zrínyi M. Chae C. H. Cho D. H. (2019). Technologies for circulating tumor cell separation from whole blood. J. Hematol. and Oncol. 12 (1), 48. 10.1186/s13045-019-0735-4 31088479 PMC6518774

[B24] Barenholz Y. (2012). Doxil® — the first FDA-approved nano-drug: lessons learned. J. Control. Release 160 (2), 117–134. 10.1016/j.jconrel.2012.03.020 22484195

[B25] Behnam M. A. Emami F. Sobhani Z. Koohi-Hosseinabadi O. Dehghanian A. R. Zebarjad S. M. (2018). Novel combination of silver nanoparticles and carbon nanotubes for plasmonic photo thermal therapy in melanoma cancer model. Adv. Pharm. Bull. 8 (1), 49–55. 10.15171/apb.2018.006 29670838 PMC5896395

[B26] Benson M. D. Waddington-Cruz M. Berk J. L. Polydefkis M. Dyck P. J. Wang A. K. (2018). Inotersen treatment for patients with hereditary transthyretin amyloidosis. N. Engl. J. Med. 379 (1), 22–31. 10.1056/nejmoa1716793 29972757 PMC12611561

[B27] Bhat A. Amanor-Boadu J. M. Guiseppi-Elie A. (2020). Toward impedimetric measurement of acidosis with a pH-responsive hydrogel sensor. ACS Sensors 5 (2), 500–509. 10.1021/acssensors.9b02336 31948224

[B28] Bhujwalla Z. M. Kakkad S. Chen Z. Jin J. Hapuarachchige S. Artemov D. (2018). Theranostics and metabolotheranostics for precision medicine in oncology. J. Magn. Reson 291, 141–151. 10.1016/j.jmr.2018.03.004 29705040 PMC5943142

[B29] Bilusic M. Madan R. A. (2012). Therapeutic cancer vaccines: the latest advancement in targeted therapy. Am. J. Ther. 19 (6), e172–e181. 10.1097/mjt.0b013e3182068cdb 21317622 PMC3601372

[B30] Biscans A. Coles A. Haraszti R. Echeverria D. Hassler M. Osborn M. (2018). Diverse lipid conjugates for functional extra-hepatic siRNA delivery *in vivo* . Nucleic Acids Res. 47 (3), 1082–1096. 10.1093/nar/gky1239 PMC6379722 30544191

[B31] Bisht S. Kanwal S. Gnanamangai B. M. Singh S. Mansi D. B. Kumar R. (2024). 3D-printed nanomedicines for cancer therapy. Future Sci. OA 10 (1), FSO973. 10.2144/fsoa-2024-0039 38817393 PMC11137762

[B32] Biswas S. Kumari P. Lakhani P. M. Ghosh B. (2016). Recent advances in polymeric micelles for anti-cancer drug delivery. Eur. J. Pharm. Sci. 83, 184–202. 10.1016/j.ejps.2015.12.031 26747018

[B33] Blass E. Ott P. A. (2021). Advances in the development of personalized neoantigen-based therapeutic cancer vaccines. Nat. Rev. Clin. Oncol. 18 (4), 215–229. 10.1038/s41571-020-00460-2 33473220 PMC7816749

[B34] Bravo-Vázquez L. A. Méndez-García A. Rodríguez A. L. Sahare P. Pathak S. Banerjee A. (2023). Applications of nanotechnologies for miRNA-based cancer therapeutics: current advances and future perspectives. Front. Bioeng. Biotechnol. 11, 1208547. 10.3389/fbioe.2023.1208547 37576994 PMC10416113

[B35] Bruni D. Angell H. K. Galon J. (2020). The immune contexture and Immunoscore in cancer prognosis and therapeutic efficacy. Nat. Rev. Cancer 20 (11), 662–680. 10.1038/s41568-020-0285-7 32753728

[B36] Bryson T. E. Anglin C. M. Bridges P. H. Cottle R. N. (2017). Nuclease-Mediated gene therapies for inherited metabolic diseases of the liver. Yale J. Biol. Med. 90 (4), 553–566. 29259521 PMC5733857

[B37] Buck J. Grossen P. Cullis P. R. Huwyler J. Witzigmann D. (2019). Lipid-based DNA therapeutics: hallmarks of non-viral gene delivery. ACS Nano 13 (4), 3754–3782. 10.1021/acsnano.8b07858 30908008

[B38] Caldeira J. C. Perrine M. Pericle F. Cavallo F. (2020). Virus-like particles as an immunogenic platform for cancer vaccines. Viruses 12 (5), 488. 10.3390/v12050488 32349216 PMC7291217

[B39] Cao Y. Huang H.-Y. Chen L.-Q. Du H.-H. Cui J.-H. Zhang L. W. (2019). Enhanced lysosomal escape of pH-responsive polyethylenimine–betaine functionalized carbon nanotube for the codelivery of survivin small interfering RNA and doxorubicin. ACS Appl. Mater. and Interfaces 11 (10), 9763–9776. 10.1021/acsami.8b20810 30776886

[B40] Chen B.-M. Cheng T.-L. Roffler S. R. (2021). Polyethylene glycol immunogenicity: theoretical, clinical, and practical aspects of anti-polyethylene glycol antibodies. ACS Nano 15 (9), 14022–14048. 10.1021/acsnano.1c05922 34469112

[B41] Chen L. Bode A. M. Dong Z. (2017b). Circulating tumor cells: moving biological insights into detection. Theranostics 7 (10), 2606–2619. 10.7150/thno.18588 28819450 PMC5558556

[B42] Chen Y. Chatterjee S. Lisok A. Minn I. Pullambhatla M. Wharram B. (2017a). A PSMA-targeted theranostic agent for photodynamic therapy. J. Photochem Photobiol. B 167, 111–116. 10.1016/j.jphotobiol.2016.12.018 28063300 PMC5310970

[B43] Chen Z. Penet M. F. Nimmagadda S. Li C. Banerjee S. R. Winnard P. T. Jr. (2012). PSMA-targeted theranostic nanoplex for prostate cancer therapy. ACS Nano 6 (9), 7752–7762. 10.1021/nn301725w 22866897 PMC4066818

[B44] Cheng M. H. Y. Overchuk M. Rajora M. A. Lou J. W. H. Chen Y. Pomper M. G. (2021b). Targeted theranostic (111)In/Lu-nanotexaphyrin for SPECT imaging and photodynamic therapy. Mol. Pharm. 19, 1803–1813. 10.1021/acs.molpharmaceut.1c00819 34965727

[B45] Cheng Y. He C. Wang M. Ma X. Mo F. Yang S. (2019). Targeting epigenetic regulators for cancer therapy: mechanisms and advances in clinical trials. Signal Transduct. Target. Ther. 4 (1), 62. 10.1038/s41392-019-0095-0 31871779 PMC6915746

[B46] Cheng Z. Li M. Dey R. Chen Y. (2021a). Nanomaterials for cancer therapy: current progress and perspectives. J. Hematol. and Oncol. 14 (1), 85. 10.1186/s13045-021-01096-0 34059100 PMC8165984

[B47] Chuah S. Chew V. (2020). High-dimensional immune-profiling in cancer: implications for immunotherapy. J. Immunother. Cancer 8 (1), e000363. 10.1136/jitc-2019-000363 32034066 PMC7057482

[B48] Chung S. Revia R. A. Zhang M. (2021). Iron oxide nanoparticles for immune cell labeling and cancer immunotherapy. Nanoscale Horizons 6 (9), 696–717. 10.1039/d1nh00179e 34286791 PMC8496976

[B49] Daramola O. O. Adara P. Adewuyi B. O. Sadiku E. R. Kupolati W. K. (2022). “3 - polymer nanoparticles (nanomedicine) for therapeutic applications,” in Polymeric biomaterials for healthcare applications. Editor Varaprasad K. (Sawston, Cambridge, England: Woodhead Publishing), 71–123.

[B50] Dasgupta I. Tanifum E. A. Srivastava M. Phatak S. S. Cavasotto C. N. Analoui M. (2012). Non inflammatory boronate based glucose-responsive insulin delivery systems. PLoS One 7 (1), e29585. 10.1371/journal.pone.0029585 22272238 PMC3260138

[B51] De Gregorio E. Rappuoli R. (2014). From empiricism to rational design: a personal perspective of the evolution of vaccine development. Nat. Rev. Immunol. 14 (7), 505–514. 10.1038/nri3694 24925139 PMC7096907

[B52] Descamps L. Le Roy D. Deman A.-L. (2022). Microfluidic-based technologies for CTC isolation: a review of 10 Years of intense efforts towards liquid biopsy. Int. J. Mol. Sci. 23 (4), 1981. 10.3390/ijms23041981 35216097 PMC8875744

[B53] Duan Y. Dhar A. Patel C. Khimani M. Neogi S. Sharma P. (2020). A brief review on solid lipid nanoparticles: part and parcel of contemporary drug delivery systems. RSC Adv. 10 (45), 26777–26791. 10.1039/d0ra03491f 35515778 PMC9055574

[B54] Eder M. Schäfer M. Bauder-Wüst U. Hull W. E. Wängler C. Mier W. (2012). 68Ga-complex lipophilicity and the targeting property of a urea-based PSMA inhibitor for PET imaging. Bioconjug Chem. 23 (4), 688–697. 10.1021/bc200279b 22369515

[B55] Eguchi A. Meade B. R. Chang Y.-C. Fredrickson C. T. Willert K. Puri N. (2009). Efficient siRNA delivery into primary cells by a peptide transduction domain–dsRNA binding domain fusion protein. Nat. Biotechnol. 27 (6), 567–571. 10.1038/nbt.1541 19448630 PMC2694965

[B56] Ekimov A. I. (1981). Quantum size effect in three-dimensional microscopic semiconductor crystals. Jetp Lett. 34, 345.

[B57] Ferdows B. E. Patel D. N. Chen W. Huang X. Kong N. Tao W. (2022). RNA cancer nanomedicine: nanotechnology-mediated RNA therapy. Nanoscale 14 (12), 4448–4455. 10.1039/d1nr06991h 35080555

[B58] Ferlay J. Colombet M. Soerjomataram I. Parkin D. M. Piñeros M. Znaor A. (2021). Cancer statistics for the year 2020: an overview. Int. J. Cancer 149 (4), 778–789. 10.1002/ijc.33588 33818764

[B59] Ferrari M. (2005). Cancer nanotechnology: opportunities and challenges. Nat. Rev. Cancer 5 (3), 161–171. 10.1038/nrc1566 15738981

[B60] Figueiras A. Domingues C. Jarak I. Santos A. I. Parra A. Pais A. (2022). New advances in biomedical application of polymeric micelles. Pharmaceutics 14 (8), 1700. 10.3390/pharmaceutics14081700 36015325 PMC9416043

[B61] Forde P. M. Spicer J. Lu S. Provencio M. Mitsudomi T. Awad M. M. (2022). Neoadjuvant nivolumab plus chemotherapy in resectable lung cancer. N. Engl. J. Med. 386 (21), 1973–1985. 10.1056/nejmoa2202170 35403841 PMC9844511

[B62] Formenti S. C. Demaria S. (2012). Radiation therapy to convert the tumor into an *in situ* vaccine. Int. J. Radiat. Oncol. Biol. Phys. 84 (4), 879–880. 10.1016/j.ijrobp.2012.06.020 23078897 PMC3811126

[B63] Frangos S. Buscombe J. R. (2019). Why should we be concerned about a “g”? Eur. J. Nucl. Med. Mol. Imaging 46 (2), 519. 10.1007/s00259-018-4204-z 30402792

[B64] Fried S. D. Boxer S. G. (2017). Electric fields and enzyme catalysis. Annu. Rev. Biochem. 86 (86), 387–415. 10.1146/annurev-biochem-061516-044432 28375745 PMC5600505

[B65] Friedlaender A. R. (2023). Oliver; meisel, alexander, PSMA-directed theragnostics: transforming prostate cancer landscape, healthbook TIMES. Oncol. Hematol. 15 (1), 16–25.

[B66] Friedrich M. Aigner A. (2022). Therapeutic siRNA: state-of-the-art and future perspectives. BioDrugs 36 (5), 549–571. 10.1007/s40259-022-00549-3 35997897 PMC9396607

[B67] Fu Z. Li S. Han S. Shi C. Zhang Y. (2022). Antibody drug conjugate: the “biological missile” for targeted cancer therapy. Signal Transduct. Target. Ther. 7 (1), 93. 10.1038/s41392-022-00947-7 35318309 PMC8941077

[B68] Galluzzi L. Buqué A. Kepp O. Zitvogel L. Kroemer G. (2017). Immunogenic cell death in cancer and infectious disease. Nat. Rev. Immunol. 17 (2), 97–111. 10.1038/nri.2016.107 27748397

[B69] Gangrade A. Mandal B. B. (2020). Drug delivery of anticancer drugs from injectable 3D porous silk scaffold for prevention of gastric cancer growth and recurrence. ACS Biomaterials Sci. and Eng. 6 (11), 6195–6206. 10.1021/acsbiomaterials.0c01043 33449660

[B70] Gao J. Karp J. M. Langer R. Joshi N. (2023). The future of drug delivery. Chem. Mater. 35 (2), 359–363. 10.1021/acs.chemmater.2c03003 37799624 PMC10553157

[B71] Garbuglia A. R. Lapa D. Sias C. Capobianchi M. R. Del Porto P. (2020). The use of both therapeutic and prophylactic vaccines in the therapy of papillomavirus disease. Front. Immunol. 11, 188. 10.3389/fimmu.2020.00188 32133000 PMC7040023

[B72] García-Uriostegui L. Meléndez-Ortíz H. I. Camacho-Villegas T. A. Lugo-Fabres P. H. Toriz G. (2022). Synthesis and characterization of mesoporous silica-g-poly(hydroxyethylmethacrylate) nanohybrid particles as a drug delivery system. Mater. Chem. Phys. 283, 126048. 10.1016/j.matchemphys.2022.126048

[B73] Garrelfs S. F. Frishberg Y. Hulton S. A. Koren M. J. O’Riordan W. D. Cochat P. (2021). Lumasiran, an RNAi therapeutic for primary hyperoxaluria type 1. N. Engl. J. Med. 384 (13), 1216–1226. 10.1056/nejmoa2021712 33789010

[B74] Gerardos A. M. Balafouti A. Pispas S. (2023). Mixed copolymer micelles for nanomedicine. Nanomanufacturing 3 (2), 233–247. 10.3390/nanomanufacturing3020015

[B75] Ghezzi M. Pescina S. Padula C. Santi P. Del Favero E. Cantù L. (2021). Polymeric micelles in drug delivery: an insight of the techniques for their characterization and assessment in biorelevant conditions. J. Control. Release 332, 312–336. 10.1016/j.jconrel.2021.02.031 33652113

[B76] Giljohann D. A. Seferos D. S. Daniel W. L. Massich M. D. Patel P. C. Mirkin C. A. (2010). Gold nanoparticles for biology and medicine. Angew. Chem. Int. Ed. 49 (19), 3280–3294. 10.1002/anie.200904359 PMC3930332 20401880

[B77] Guiseppi-Elie A. Brahim S. I. Narinesingh D. (2002). A chemically synthesized artificial pancreas: release of insulin from glucose-responsive hydrogels. Adv. Mater. 14 (10), 743–746. 10.1002/1521-4095(20020517)14:10<743::aid-adma743>3.0.co;2-h

[B78] Guo Y. Wang Z. Shi X. Shen M. (2022). Engineered cancer cell membranes: an emerging agent for efficient cancer theranostics. Exploration 2 (1), 20210171. 10.1002/exp.20210171 37324583 PMC10190949

[B79] Habli Z. AlChamaa W. Saab R. Kadara H. Khraiche M. L. (2020). Circulating tumor cell detection technologies and clinical utility: challenges and opportunities. Cancers 12 (7), 1930. 10.3390/cancers12071930 32708837 PMC7409125

[B80] Haider M. S. Lübtow M. M. Endres S. Forster S. Flegler V. J. Böttcher B. (2020). Think beyond the core: impact of the hydrophilic corona on drug solubilization using polymer micelles. ACS Appl. Mater. and Interfaces 12 (22), 24531–24543. 10.1021/acsami.9b22495 32378873

[B81] Halder J. Pradhan D. Biswasroy P. Rai V. K. Kar B. Ghosh G. (2022). Trends in iron oxide nanoparticles: a nano-platform for theranostic application in breast cancer. J. Drug Target. 30 (10), 1055–1075. 10.1080/1061186x.2022.2095389 35786242

[B82] Hammer S. Hagemann U. B. Zitzmann-Kolbe S. Larsen A. Ellingsen C. Geraudie S. (2020). Preclinical efficacy of a PSMA-targeted thorium-227 conjugate (PSMA-TTC), a targeted alpha therapy for prostate cancer. Clin. Cancer Res. 26 (8), 1985–1996. 10.1158/1078-0432.ccr-19-2268 31831560

[B83] Hammond S. M. Sergeeva O. V. Melnikov P. A. Goli L. Stoodley J. Zatsepin T. S. (2021). Mesyl phosphoramidate oligonucleotides as potential splice-switching agents: impact of backbone structure on activity and intracellular localization. Nucleic Acid. Ther. 31 (3), 190–200. 10.1089/nat.2020.0860 33989066

[B84] Hazra R. S. Dutta D. Mamnoon B. Nair G. Knight A. Mallik S. (2021). Polymeric composite matrix with high biobased content as pharmaceutically relevant molecular encapsulation and release platform. ACS Appl. Mater. and Interfaces 13 (34), 40229–40248. 10.1021/acsami.1c03805 34423963

[B85] Hazra R. S. Kale N. Aland G. Qayyumi B. Mitra D. Jiang L. (2020). Cellulose mediated transferrin nanocages for enumeration of circulating tumor cells for head and neck cancer. Sci. Rep. 10 (1), 10010. 10.1038/s41598-020-66625-2 32561829 PMC7305211

[B86] Hazra R. S. Kale N. Boyle C. Molina K. B. D'Souza A. Aland G. (2024). Magnetically-activated, nanostructured cellulose for efficient capture of circulating tumor cells from the blood sample of head and neck cancer patients. Carbohydr. Polym. 323, 121418. 10.1016/j.carbpol.2023.121418 37940250

[B87] Heegaard P. M. H. Boas U. Sorensen N. S. (2010). Dendrimers for vaccine and immunostimulatory uses. A review. Bioconjugate Chem. 21 (3), 405–418. 10.1021/bc900290d 19886668

[B88] Hou X. Shen G. Zhou L. Li Y. Wang T. Ma X. (2022). Artificial intelligence in cervical cancer screening and diagnosis. Front. Oncol. 12, 851367. 10.3389/fonc.2022.851367 35359358 PMC8963491

[B89] Howlader N. Forjaz G. Mooradian M. J. Meza R. Kong C. Y. Cronin K. A. (2020). The effect of advances in lung-cancer treatment on population mortality. N. Engl. J. Med. 383 (7), 640–649. 10.1056/nejmoa1916623 32786189 PMC8577315

[B90] Huang L. Li Y. Du Y. Zhang Y. Wang X. Ding Y. (2019). Mild photothermal therapy potentiates anti-PD-L1 treatment for immunologically cold tumors via an all-in-one and all-in-control strategy. Nat. Commun. 10 (1), 4871. 10.1038/s41467-019-12771-9 31653838 PMC6814770

[B91] Huang L. Zhang Y. Li Y. Meng F. Li H. Zhang H. (2021). Time-programmed delivery of sorafenib and anti-CD47 antibody via a double-layer-gel matrix for postsurgical treatment of breast cancer. Nano-Micro Lett. 13 (1), 141. 10.1007/s40820-021-00647-x PMC8197688 34138357

[B92] Huang W. Pang Y. Liu Q. Liang C. An S. Wu Q. (2023). Development and characterization of novel FAP-targeted theranostic pairs: a bench-to-bedside study. Research 6, 0282. 10.34133/research.0282 38706713 PMC11066877

[B93] Huynh C. T. Nguyen M. K. Tonga G. Y. Longé L. Rotello V. M. Alsberg E. (2016). Photocleavable hydrogels for light-triggered siRNA release. Adv. Healthc. Mater. 5 (3), 305–310. 10.1002/adhm.201500778 26639103 PMC4755586

[B94] Huynh C. T. Zheng Z. Nguyen M. K. McMillan A. Yesilbag Tonga G. Rotello V. M. (2017). Cytocompatible catalyst-free photodegradable hydrogels for light-mediated RNA release to induce hMSC osteogenesis. ACS Biomaterials Sci. and Eng. 3 (9), 2011–2023. 10.1021/acsbiomaterials.6b00796 PMC13380960 33440556

[B95] Iglesias P. A. Ingalls B. P. (2010). Control theory and systems biology. Cambridge, Mass: MIT Press.

[B96] Jacques Ferlay M. C. Soerjomataram I. Parkin D. M. Piñeros M. Znaor A. Bray F. (2020). Global cancer observatory: cancer today. Lyon, France: WHO International Agency for Research on Cancer.

[B97] Jana J. Ganguly M. Pal T. (2016). Enlightening surface plasmon resonance effect of metal nanoparticles for practical spectroscopic application. RSC Adv. 6 (89), 86174–86211. 10.1039/c6ra14173k

[B98] Janko C. Ratschker T. Nguyen K. Zschiesche L. Tietze R. Lyer S. (2019). Functionalized superparamagnetic iron oxide nanoparticles (SPIONs) as platform for the targeted multimodal tumor therapy. Front. Oncol. 9, 59. 10.3389/fonc.2019.00059 30815389 PMC6382019

[B99] Jarrett A. M. Faghihi D. Hormuth D. A. Lima E. A. B. F. Virostko J. Biros G. (2020). Optimal control theory for personalized therapeutic regimens in oncology: background, history, challenges, and opportunities. J. Clin. Med. 9 (5), 1314. 10.3390/jcm9051314 32370195 PMC7290915

[B100] Jhunjhunwala S. Hammer C. Delamarre L. (2021). Antigen presentation in cancer: insights into tumour immunogenicity and immune evasion. Nat. Rev. Cancer 21 (5), 298–312. 10.1038/s41568-021-00339-z 33750922

[B101] Jin L. Zhao W. Zhang J. Chen W. Xie T. Wang L. (2020). Evaluation of the diagnostic value of circulating tumor cells with CytoSorter^®^ CTC capture system in patients with breast cancer. Cancer Med. 9 (5), 1638–1647. 10.1002/cam4.2825 31908156 PMC7050089

[B102] Juan C. A. Pérez de la Lastra J. M. Plou F. J. Pérez-Lebeña E. (2021). The chemistry of reactive oxygen species (ROS) revisited: outlining their role in biological macromolecules (DNA, lipids and proteins) and induced pathologies. Int. J. Mol. Sci. 22 (9), 4642. 10.3390/ijms22094642 33924958 PMC8125527

[B103] Justyna Frączyk M. W. Balcerzak W. Pokajewicz K. Wieczorek P. Młynarski W. Fendler W. (2018). Kamiński Molecular fingerprinting of thyroid cancer cells using library of molecular receptors formed by n-lipidated peptides immobilized on cellulose. Acta Poloniae Pharm. - Drug Res. 75 (4), 1017–1029.

[B104] Kaewput C. Vinjamuri S. (2022). Update of PSMA theranostics in prostate cancer: current applications and future trends. J. Clin. Med. 11 (10), 2738. 10.3390/jcm11102738 35628867 PMC9144463

[B105] Kamath V. (2021). Cancer vaccines: an unkept promise? Drug Discov. Today 26 (6), 1347–1352. 10.1016/j.drudis.2021.02.006 33601016

[B106] Karathanasis E. Bhavane R. Annapragada A. V. (2007). Glucose-sensing pulmonary delivery of human insulin to the systemic circulation of rats. Int. J. Nanomedicine 2 (3), 501–513. 18019848 PMC2676664

[B107] Kelly J. Amor-Coarasa A. Ponnala S. Nikolopoulou A. Williams C. Jr. Schlyer D. (2018). Trifunctional PSMA-targeting constructs for prostate cancer with unprecedented localization to LNCaP tumors. Eur. J. Nucl. Med. Mol. Imaging 45 (11), 1841–1851. 10.1007/s00259-018-4004-5 29623376

[B108] Kelly J. M. Ponnala S. Amor-Coarasa A. Zia N. A. Nikolopoulou A. Williams C. Jr. (2020). Preclinical evaluation of a high-affinity sarcophagine-containing PSMA ligand for 64Cu/67Cu-based theranostics in prostate cancer. Mol. Pharm. 17 (6), 1954–1962. 10.1021/acs.molpharmaceut.0c00060 32286841

[B109] Kemp J. A. Kwon Y. J. (2021). Cancer nanotechnology: current status and perspectives. Nano Converg. 8 (1), 34. 10.1186/s40580-021-00282-7 34727233 PMC8560887

[B110] Kerr M. D. McBride D. A. Chumber A. K. Shah N. J. (2021). Combining therapeutic vaccines with chemo- and immunotherapies in the treatment of cancer. Expert Opin. Drug Discov. 16 (1), 89–99. 10.1080/17460441.2020.1811673 32867561 PMC7785654

[B111] Khan T. Weber H. DiMuzio J. Matter A. Dogdas B. Shah T. (2016). Silencing myostatin using cholesterol-conjugated siRNAs induces muscle growth. Mol. Ther. - Nucleic Acids 5, e342. 10.1038/mtna.2016.55 27483025 PMC5023400

[B112] Kim J. J. Park K. (2001). Modulated insulin delivery from glucose-sensitive hydrogel dosage forms. J. Control. Release 77 (1), 39–47. 10.1016/s0168-3659(01)00447-3 11689258

[B113] Kim S. Choi Y. Kim K. (2022). Coacervate-mediated novel pancreatic cancer drug Aleuria Aurantia lectin delivery for augmented anticancer therapy. Biomaterials Res. 26 (1), 35. 10.1186/s40824-022-00282-6 PMC9308356 35869562

[B114] Kim Y.-K. (2020). RNA therapy: current status and future potential. Chonnam Med. J. 56 (2), 87–93. 10.4068/cmj.2020.56.2.87 32509554 PMC7250668

[B115] Kinali M. Arechavala-Gomeza V. Feng L. Cirak S. Hunt D. Adkin C. (2009). Local restoration of dystrophin expression with the morpholino oligomer AVI-4658 in Duchenne muscular dystrophy: a single-blind, placebo-controlled, dose-escalation, proof-of-concept study. Lancet Neurology 8 (10), 918–928. 10.1016/s1474-4422(09)70211-x 19713152 PMC2755039

[B116] Kocak G. Tuncer C. Bütün V. (2017). pH-Responsive polymers. Polym. Chem. 8 (1), 144–176. 10.1039/c6py01872f

[B117] Koch B. Rubino I. Quan F.-S. Yoo B. Choi H.-J. (2016). Microfabrication for drug delivery. Materials 9 (8), 646. 10.3390/ma9080646 28773770 PMC5509096

[B118] Koh D.-M. Papanikolaou N. Bick U. Illing R. Kahn C. E. Kalpathi-Cramer J. (2022). Artificial intelligence and machine learning in cancer imaging. Commun. Med. 2 (1), 133. 10.1038/s43856-022-00199-0 36310650 PMC9613681

[B119] Krajcik R. Jung A. Hirsch A. Neuhuber W. Zolk O. (2008). Functionalization of carbon nanotubes enables non-covalent binding and intracellular delivery of small interfering RNA for efficient knock-down of genes. Biochem. Biophysical Res. Commun. 369 (2), 595–602. 10.1016/j.bbrc.2008.02.072 18298946

[B120] Kunze D. Erdmann K. Froehner M. Wirth M. P. Fuessel S. (2012). siRNA-mediated inhibition of antiapoptotic genes enhances chemotherapy efficacy in bladder cancer cells. Anticancer Res. 32 (10), 4313–4318. 23060552

[B121] Lamb Y. N. (2021). Inclisiran: first approval. Drugs 81 (3), 389–395. 10.1007/s40265-021-01473-6 33620677 PMC7900795

[B122] Lawrence R. Watters M. Davies C. R. Pantel K. Lu Y.-J. (2023). Circulating tumour cells for early detection of clinically relevant cancer. Nat. Rev. Clin. Oncol. 20 (7), 487–500. 10.1038/s41571-023-00781-y 37268719 PMC10237083

[B123] Le N. Kim K. (2023). Current advances in the biomedical applications of quantum dots: promises and challenges. Int. J. Mol. Sci. 24 (16), 12682. 10.3390/ijms241612682 37628860 PMC10454335

[B124] Lecca P. (2021). Control theory and cancer chemotherapy: how they interact. Front. Bioeng. Biotechnol. 8, 621269. 10.3389/fbioe.2020.621269 33520972 PMC7841331

[B125] LeDuc P. R. Messner W. C. Wikswo J. P. (2011). How do control-based approaches enter into biology? Annu. Rev. Biomed. Eng. 13 (1), 369–396. 10.1146/annurev-bioeng-071910-124651 21599491

[B126] Lee C.-F. Yang C.-H. Lin T.-L. Bahadur P. Chen L.-J. (2019). Role of molecular weight and hydrophobicity of amphiphilic tri-block copolymers in temperature-dependent co-micellization process and drug solubility. Colloids Surfaces B Biointerfaces 183, 110461. 10.1016/j.colsurfb.2019.110461 31479972

[B127] Lee W. Im H. J. (2019). Theranostics based on liposome: looking back and forward. Nucl. Med. Mol. Imaging 53 (4), 242–246. 10.1007/s13139-019-00603-z 31456856 PMC6694360

[B128] Li F. Xu H. Zhao Y. (2021). Magnetic particles as promising circulating tumor cell catchers assisting liquid biopsy in cancer diagnosis: a review. TrAC Trends Anal. Chem. 145, 116453. 10.1016/j.trac.2021.116453

[B129] Li L. Hou J. Liu X. Guo Y. Wu Y. Zhang L. (2014). Nucleolin-targeting liposomes guided by aptamer AS1411 for the delivery of siRNA for the treatment of malignant melanomas. Biomaterials 35 (12), 3840–3850. 10.1016/j.biomaterials.2014.01.019 24486214

[B130] Li L. Yang W.-W. Xu D.-G. (2019). Stimuli-responsive nanoscale drug delivery systems for cancer therapy. J. Drug Target. 27 (4), 423–433. 10.1080/1061186x.2018.1519029 30173577

[B131] Li M. Zhu J. Lv Z. Qin H. Wang X. Shi H. (2024). Recent advances in RNA-targeted cancer therapy. ChemBioChem 25 (4), e202300633. 10.1002/cbic.202300633 37961028

[B132] Li Z. B. Wu Z. Chen K. Ryu E. K. Chen X. (2008). 18F-labeled BBN-RGD heterodimer for prostate cancer imaging. J. Nucl. Med. 49 (3), 453–461. 10.2967/jnumed.107.048009 18287274

[B133] Liang X. Li D. Leng S. Zhu X. (2020). RNA-based pharmacotherapy for tumors: from bench to clinic and back. Biomed. and Pharmacother. 125, 109997. 10.1016/j.biopha.2020.109997 32062550

[B134] Lin M. J. Svensson-Arvelund J. Lubitz G. S. Marabelle A. Melero I. Brown B. D. (2022). Cancer vaccines: the next immunotherapy frontier. Nat. Cancer 3 (8), 911–926. 10.1038/s43018-022-00418-6 35999309

[B135] Lin X. Song X. Zhang Y. Cao Y. Xue Y. Wu F. (2020). Multifunctional theranostic nanosystems enabling photothermal-chemo combination therapy of triple-stimuli-responsive drug release with magnetic resonance imaging. Biomater. Sci. 8 (7), 1875–1884. 10.1039/c9bm01482a 32010912

[B136] Liu H. Nie T. Duan X. Zhang X. Zheng Y. Zhong W. (2023). Cerebral delivery of redox-responsive lenalidomide prodrug plus methotrexate for primary central nerve system lymphoma combination therapy. J. Control. Release 359, 132–146. 10.1016/j.jconrel.2023.05.040 37269965

[B137] Liu K. Jing B. Kang J. Han L. Chang J. (2024). Ultrasound-enabled nanomedicine for tumor theranostics. Engineering. 10.1016/j.eng.2024.01.030

[B138] Liu X. Wang W. Samarsky D. Liu L. Xu Q. Zhang W. (2014). Tumor-targeted *in vivo* gene silencing via systemic delivery of cRGD-conjugated siRNA. Nucleic Acids Res. 42 (18), 11805–11817. 10.1093/nar/gku831 25223783 PMC4191406

[B139] Liu Y. Hardie J. Zhang X. Rotello V. M. (2017). Effects of engineered nanoparticles on the innate immune system. Semin. Immunol. 34, 25–32. 10.1016/j.smim.2017.09.011 28985993 PMC5705289

[B140] Liu Y. Yan X. Zhang F. Zhang X. Tang F. Han Z. (2021). TCR-T immunotherapy: the challenges and solutions. Front. Oncol. 11, 794183. 10.3389/fonc.2021.794183 35145905 PMC8822241

[B141] Liu Z. Ran H. Wang Z. Zhou S. Wang Y. (2019). Targeted and pH-facilitated theranostic of orthotopic gastric cancer via phase-transformation doxorubicin-encapsulated nanoparticles enhanced by low-intensity focused ultrasound (LIFU) with reduced side effect. Int. J. Nanomedicine 14, 7627–7642. 10.2147/ijn.s212888 31571868 PMC6757192

[B142] Lollini P.-L. Cavallo F. Nanni P. Forni G. (2006). Vaccines for tumour prevention. Nat. Rev. Cancer 6 (3), 204–216. 10.1038/nrc1815 16498443

[B143] Lorenz C. Hadwiger P. John M. Vornlocher H.-P. Unverzagt C. (2004). Steroid and lipid conjugates of siRNAs to enhance cellular uptake and gene silencing in liver cells. Bioorg. and Med. Chem. Lett. 14 (19), 4975–4977. 10.1016/j.bmcl.2004.07.018 15341962

[B144] Lyu Z. Ding L. Tintaru A. Peng L. (2020). Self-assembling supramolecular dendrimers for biomedical applications: lessons learned from poly(amidoamine) dendrimers. Acc. Chem. Res. 53 (12), 2936–2949. 10.1021/acs.accounts.0c00589 33275845

[B145] Ma P. Lai X. Luo Z. Chen Y. Loh X. J. Ye E. (2022). Recent advances in mechanical force-responsive drug delivery systems. Nanoscale Adv. 4 (17), 3462–3478. 10.1039/d2na00420h 36134346 PMC9400598

[B146] Madan R. A. Antonarakis E. S. Drake C. G. Fong L. Yu E. Y. McNeel D. G. (2020). Putting the pieces together: completing the mechanism of action jigsaw for sipuleucel-T. JNCI J. Natl. Cancer Inst. 112 (6), 562–573. 10.1093/jnci/djaa021 32145020 PMC7301097

[B147] Majumder J. Minko T. (2021). Multifunctional and stimuli-responsive nanocarriers for targeted therapeutic delivery. Expert Opin. Drug Deliv. 18 (2), 205–227. 10.1080/17425247.2021.1828339 32969740 PMC7904578

[B148] Malekzadeh P. Yossef R. Cafri G. Paria B. C. Lowery F. J. Jafferji M. (2020). Antigen experienced T cells from peripheral blood recognize p53 neoantigens. Clin. Cancer Res. 26 (6), 1267–1276. 10.1158/1078-0432.ccr-19-1874 31996390 PMC7424598

[B149] Maly V. Maly O. Kolostova K. Bobek V. (2019). Circulating tumor cells in diagnosis and treatment of lung cancer. Vivo 33 (4), 1027–1037. 10.21873/invivo.11571 PMC6689346 31280190

[B150] Mansoori B. Sandoghchian Shotorbani S. Baradaran B. (2014). RNA interference and its role in cancer therapy. Adv. Pharm. Bull. 4 (4), 313–321. 10.5681/apb.2014.046 25436185 PMC4137419

[B151] Massoud T. F. Gambhir S. S. (2003). Molecular imaging in living subjects: seeing fundamental biological processes in a new light. Genes Dev. 17 (5), 545–580. 10.1101/gad.1047403 12629038

[B152] Matsuda S. Keiser K. Nair J. K. Charisse K. Manoharan R. M. Kretschmer P. (2015). siRNA conjugates carrying sequentially assembled trivalent N-acetylgalactosamine linked through nucleosides elicit robust gene silencing *in vivo* in hepatocytes. ACS Chem. Biol. 10 (5), 1181–1187. 10.1021/cb501028c 25730476

[B153] Matsuda T. Cepko C. L. (2004). Electroporation and RNA interference in the rodent retina *in vivo* and *in vitro* . Proc. Natl. Acad. Sci. 101 (1), 16–22. 10.1073/pnas.2235688100 14603031 PMC314130

[B154] Mazidi Z. Javanmardi S. Naghib S. M. Mohammadpour Z. (2022). Smart stimuli-responsive implantable drug delivery systems for programmed and on-demand cancer treatment: an overview on the emerging materials. Chem. Eng. J. 433, 134569. 10.1016/j.cej.2022.134569

[B155] McHugh K. J. Jing L. Severt S. Y. Cruz M. Sarmadi M. Jayawardena H. S. N. (2019). Biocompatible near-infrared quantum dots delivered to the skin by microneedle patches record vaccination. Sci. Transl. Med. 11 (523), eaay7162. 10.1126/scitranslmed.aay7162 31852802 PMC7532118

[B156] McInnes L. E. Cullinane C. Roselt P. D. Jackson S. Blyth B. J. Dam E. M. v. (2021). Therapeutic efficacy of a bivalent inhibitor of prostate-specific membrane antigen labeled with ^67^Cu. J. Nucl. Med. 62 (6), 829–832. 10.2967/jnumed.120.251579 33067341 PMC8729863

[B157] McNamara J. O. Andrechek E. R. Wang Y. Viles K. D. Rempel R. E. Gilboa E. (2006). Cell type–specific delivery of siRNAs with aptamer-siRNA chimeras. Nat. Biotechnol. 24 (8), 1005–1015. 10.1038/nbt1223 16823371

[B158] Melero I. Berman D. M. Aznar M. A. Korman A. J. Pérez Gracia J. L. Haanen J. (2015). Evolving synergistic combinations of targeted immunotherapies to combat cancer. Nat. Rev. Cancer 15 (8), 457–472. 10.1038/nrc3973 26205340

[B159] Miroshnichenko S. K. Patutina O. A. Burakova E. A. Chelobanov B. P. Fokina A. A. Vlassov V. V. (2019). Mesyl phosphoramidate antisense oligonucleotides as an alternative to phosphorothioates with improved biochemical and biological properties. Proc. Natl. Acad. Sci. 116 (4), 1229–1234. 10.1073/pnas.1813376116 30622178 PMC6347720

[B160] Mitchell M. J. Billingsley M. M. Haley R. M. Wechsler M. E. Peppas N. A. Langer R. (2021). Engineering precision nanoparticles for drug delivery. Nat. Rev. Drug Discov. 20 (2), 101–124. 10.1038/s41573-020-0090-8 33277608 PMC7717100

[B161] Mohebian Z. Babazadeh M. Zarghami N. Mousazadeh H. (2021). Anticancer efficiency of curcumin-loaded mesoporous silica nanoparticles/nanofiber composites for potential postsurgical breast cancer treatment. J. Drug Deliv. Sci. Technol. 61, 102170. 10.1016/j.jddst.2020.102170

[B162] Mohsen M. O. Bachmann M. F. (2022). Virus-like particle vaccinology, from bench to bedside. Cell. and Mol. Immunol. 19 (9), 993–1011. 10.1038/s41423-022-00897-8 35962190 PMC9371956

[B163] Mondal A. Nayak A. K. Chakraborty P. Banerjee S. Nandy B. C. (2023). Natural polymeric nanobiocomposites for anti-cancer drug delivery therapeutics: a recent update. Pharmaceutics 15 (8), 2064. 10.3390/pharmaceutics15082064 37631276 PMC10459560

[B164] Müller C. Umbricht C. A. Gracheva N. Tschan V. J. Pellegrini G. Bernhardt P. (2019). Terbium-161 for PSMA-targeted radionuclide therapy of prostate cancer. Eur. J. Nucl. Med. Mol. Imaging 46 (9), 1919–1930. 10.1007/s00259-019-04345-0 31134301 PMC6820371

[B165] Muluneh M. Issadore D. (2014). Microchip-based detection of magnetically labeled cancer biomarkers. Adv. Drug Deliv. Rev. 66, 101–109. 10.1016/j.addr.2013.09.013 24099664 PMC4418637

[B166] Muñoz-López P. Ribas-Aparicio R. M. Becerra-Báez E. I. Fraga-Pérez K. Flores-Martínez L. F. Mateos-Chávez A. A. (2022). Single-chain fragment variable: recent progress in cancer diagnosis and therapy. Cancers (Basel) 14 (17), 4206. 10.3390/cancers14174206 36077739 PMC9455005

[B167] Muramatsu T. Nakamura A. Park H. M. (1998). *In vivo* electroporation: a powerful and convenient means of nonviral gene transfer to tissues of living animals (Review). Int. J. Mol. Med. 1 (1), 55–62. 10.3892/ijmm.1.1.55 9852198

[B168] Murray C. Norris D. J. Bawendi M. G. (1993). Synthesis and characterization of nearly monodisperse CdE (E= sulfur, selenium, tellurium) semiconductor nanocrystallites. J. Am. Chem. Soc. 115 (19), 8706–8715. 10.1021/ja00072a025

[B169] Naletova I. Tomasello B. Attanasio F. Pleshkan V. V. (2023). Prospects for the use of metal-based nanoparticles as adjuvants for local cancer immunotherapy. Pharmaceutics 15 (5), 1346. 10.3390/pharmaceutics15051346 37242588 PMC10222518

[B170] Nel J. Elkhoury K. Velot É. Bianchi A. Acherar S. Francius G. (2023). Functionalized liposomes for targeted breast cancer drug delivery. Bioact. Mater. 24, 401–437. 10.1016/j.bioactmat.2022.12.027 36632508 PMC9812688

[B171] Ng E. W. M. Shima D. T. Calias P. Cunningham E. T. Guyer D. R. Adamis A. P. (2006). Pegaptanib, a targeted anti-VEGF aptamer for ocular vascular disease. Nat. Rev. Drug Discov. 5 (2), 123–132. 10.1038/nrd1955 16518379

[B172] Nie T. Liu H. Fang Z. Zheng Y. Zhang R. Xu X. (2023). Tumor microenvironment mediated spermidine-metal-immunopeptide nanocomplex for boosting ferroptotic immunotherapy of lymphoma. ACS Nano 17 (11), 10925–10937. 10.1021/acsnano.3c02803 37219600

[B173] Nikan M. Osborn M. F. Coles A. H. Godinho B. M. D. C. Hall L. M. Haraszti R. A. (2016). Docosahexaenoic acid conjugation enhances distribution and safety of siRNA upon local administration in mouse brain. Mol. Ther. - Nucleic Acids 5, e344. 10.1038/mtna.2016.50 27504598 PMC5023396

[B174] Nooraei S. Bahrulolum H. Hoseini Z. S. Katalani C. Hajizade A. Easton A. J. (2021). Virus-like particles: preparation, immunogenicity and their roles as nanovaccines and drug nanocarriers. J. Nanobiotechnology 19 (1), 59. 10.1186/s12951-021-00806-7 33632278 PMC7905985

[B175] Novoselova M. V. Shramova E. I. Sergeeva O. V. Shcherbinina E. Y. Perevoschikov S. V. Melnikov P. (2023). Polymer/magnetite carriers functionalized by HER2-DARPin: avoiding lysosomes during internalization and controlled toxicity of doxorubicin by focused ultrasound induced release. Nanomedicine 47, 102612. 10.1016/j.nano.2022.102612 36243307

[B176] Olvera D. Monaghan M. G. (2021). Electroactive material-based biosensors for detection and drug delivery. Adv. Drug Deliv. Rev. 170, 396–424. 10.1016/j.addr.2020.09.011 32987096

[B177] Paciotti G. F. Kingston D. G. I. Tamarkin L. (2006). Colloidal gold nanoparticles: a novel nanoparticle platform for developing multifunctional tumor-targeted drug delivery vectors. Drug Dev. Res. 67 (1), 47–54. 10.1002/ddr.20066

[B178] Pan P. Svirskis D. Rees S. W. P. Barker D. Waterhouse G. I. N. Wu Z. (2021). Photosensitive drug delivery systems for cancer therapy: mechanisms and applications. J. Control. Release 338, 446–461. 10.1016/j.jconrel.2021.08.053 34481021

[B179] Pan X. Huang W. Nie G. Wang C. Wang H. (2024). Ultrasound-sensitive intelligent nanosystems: a promising strategy for the treatment of neurological diseases. Adv. Mater. 36 (22), 2303180. 10.1002/adma.202303180 37871967

[B180] Patra A. Satpathy S. Shenoy A. K. Bush J. A. Kazi M. Hussain M. D. (2018). Formulation and evaluation of mixed polymeric micelles of quercetin for treatment of breast, ovarian, and multidrug resistant cancers. Int. J. Nanomedicine 13, 2869–2881. 10.2147/ijn.s153094 29844670 PMC5961470

[B181] Patrick B. Akhtar T. Kousar R. Huang C.-C. Li X.-G. (2023). Carbon nanomaterials: emerging roles in immuno-oncology. Int. J. Mol. Sci. 24 (7), 6600. 10.3390/ijms24076600 37047572 PMC10095276

[B182] Paul A. Muralidharan A. Biswas A. Kamath B. V. Joseph A. Alex A. T. (2022). siRNA therapeutics and its challenges: recent advances in effective delivery for cancer therapy. OpenNano 7, 100063. 10.1016/j.onano.2022.100063

[B183] Paunovska K. Loughrey D. Dahlman J. E. (2022). Drug delivery systems for RNA therapeutics. Nat. Rev. Genet. 23 (5), 265–280. 10.1038/s41576-021-00439-4 34983972 PMC8724758

[B184] Perotti M. Perez L. (2019). Virus-like particles and nanoparticles for vaccine development against HCMV. Viruses 12 (1), 35. 10.3390/v12010035 31905677 PMC7019358

[B185] Pham S. H. Choi Y. Choi J. (2020). Stimuli-responsive nanomaterials for application in antitumor therapy and drug delivery. Pharmaceutics 12 (7), 630. 10.3390/pharmaceutics12070630 32635539 PMC7408499

[B186] Pirmoradi F. N. Chiao M. (2013). “Reservoir-based MEMS drug delivery system,” in Encyclopedia of microfluidics and nanofluidics. Editor Li D. (US, Boston, MA: Springer), 1–7.

[B187] Polakova I. Duskova M. Smahel M. (2014). Antitumor DNA vaccination against the Sox2 transcription factor. Int. J. Oncol. 45 (1), 139–146. 10.3892/ijo.2014.2402 24789529

[B265] Porciuncula A. Morgado M. Gupta R. Syrigos K. Meehan R. Zacharek S. J. (2021). Spatial mapping and immunomodulatory role of the OX40/OX40L pathway in human non–small cell lung cancer. Clin. Cancer Res. 27 (22), 6174–6183. 10.1158/1078-0432.CCR-21-0987 34518312 PMC8595671

[B188] Rahim M. A. Jan N. Khan S. Shah H. Madni A. Khan A. (2021). Recent advancements in stimuli responsive drug delivery platforms for active and passive cancer targeting. Cancers (Basel) 13 (4), 670. 10.3390/cancers13040670 33562376 PMC7914759

[B189] Rapp T. L. DeForest C. A. (2021). Targeting drug delivery with light: a highly focused approach. Adv. Drug Deliv. Rev. 171, 94–107. 10.1016/j.addr.2021.01.009 33486009 PMC8127392

[B190] Roces C. B. Lou G. Jain N. Abraham S. Thomas A. Halbert G. W. (2020). Manufacturing considerations for the development of lipid nanoparticles using microfluidics. Pharmaceutics 12 (11), 1095. 10.3390/pharmaceutics12111095 33203082 PMC7697682

[B191] Roelofsen L. M. Kaptein P. Thommen D. S. (2022). Multimodal predictors for precision immunotherapy. Immuno-Oncology Technol. 14, 100071. 10.1016/j.iotech.2022.100071 PMC9216437 35755892

[B192] Rosenblum H. G. Lewis R. M. Gargano J. W. Querec T. D. Unger E. R. Markowitz L. E. (2022). Human papillomavirus vaccine impact and effectiveness through 12 Years after vaccine introduction in the United States, 2003 to 2018. Ann. Intern Med. 175 (7), 918–926. 10.7326/m21-3798 35576590 PMC11614147

[B193] Rossetti R. Brus L. (1982). Electron-hole recombination emission as a probe of surface chemistry in aqueous cadmium sulfide colloids. J. Phys. Chem. 86 (23), 4470–4472. 10.1021/j100220a003

[B194] Rudloff M. W. Zumbo P. Favret N. R. Roetman J. J. Detrés Román C. R. Erwin M. M. (2023). Hallmarks of CD8+ T cell dysfunction are established within hours of tumor antigen encounter before cell division. Nat. Immunol. 24 (9), 1527–1539. 10.1038/s41590-023-01578-y 37537361 PMC10878719

[B195] Rühle J. Klemt I. Abakumova T. Sergeeva O. Vetosheva P. Zatsepin T. (2022). Reactive oxygen species-responsive RNA interference. Chem. Commun. 58 (27), 4388–4391. 10.1039/d2cc00651k 35297916

[B196] Saleh R. Taha R. Z. Toor S. M. Sasidharan Nair V. Murshed K. Khawar M. (2020). Expression of immune checkpoints and T cell exhaustion markers in early and advanced stages of colorectal cancer. Cancer Immunol. Immunother. 69 (10), 1989–1999. 10.1007/s00262-020-02593-w 32393998 PMC7511277

[B197] Salvador M. Gutiérrez G. Noriega S. Moyano A. Blanco-López M. C. Matos M. (2021). Microemulsion synthesis of superparamagnetic nanoparticles for bioapplications. Int. J. Mol. Sci. 22 (1), 427. 10.3390/ijms22010427 33406682 PMC7795751

[B198] Samanta D. Hosseini-Nassab N. Zare R. N. (2016). Electroresponsive nanoparticles for drug delivery on demand. Nanoscale 8 (17), 9310–9317. 10.1039/c6nr01884j 27088543

[B199] Schoenmaker L. Witzigmann D. Kulkarni J. A. Verbeke R. Kersten G. Jiskoot W. (2021). mRNA-lipid nanoparticle COVID-19 vaccines: structure and stability. Int. J. Pharm. 601, 120586. 10.1016/j.ijpharm.2021.120586 33839230 PMC8032477

[B200] Şen Karaman D. Kettiger H. (2018). “Chapter 1 - silica-based nanoparticles as drug delivery systems: chances and challenges,” in Inorganic frameworks as smart nanomedicines. Editor Grumezescu A. M. (Norwich, NY: William Andrew Publishing), 1–40.

[B201] Shi Z. Hu Y. Li X. (2024). Polymer mechanochemistry in drug delivery: from controlled release to precise activation. J. Control. Release 365, 259–273. 10.1016/j.jconrel.2023.10.042 39491171

[B202] Shreve M. J. T. Khanani M. S. A. Haddad M. T. C. (2022). Artificial intelligence in oncology: current capabilities, future opportunities, and ethical considerations. Am. Soc. Clin. Oncol. Educ. Book 42 (42), 1–10. 10.1200/edbk_350652 35687826

[B203] Siegel R. L. Miller K. D. Wagle N. S. Jemal A. (2023). Cancer statistics. CA A Cancer J. Clin. 73 (1), 17–48. 10.3322/caac.21763 36633525

[B204] Singh B. Arora S. D’Souza A. Kale N. Aland G. Bharde A. (2021). Chemo-specific designs for the enumeration of circulating tumor cells: advances in liquid biopsy. J. Mater. Chem. B 9 (13), 2946–2978. 10.1039/d0tb02574g 33480960

[B205] Smith P. L. Piadel K. Dalgleish A. G. (2021). Directing T-cell immune responses for cancer vaccination and immunotherapy. Vaccines (Basel) 9 (12), 1392. 10.3390/vaccines9121392 34960140 PMC8708201

[B206] Sobhani Z. Behnam M. A. Emami F. Dehghanian A. Jamhiri I. (2017). Photothermal therapy of melanoma tumor using multiwalled carbon nanotubes. Int. J. Nanomedicine 12, 4509–4517. 10.2147/ijn.s134661 28684911 PMC5484561

[B207] Sobol I. Thompson R. H. Dong H. Krco C. Kwon E. D. (2015). Immunotherapy in prostate cancer. Curr. Urol. Rep. 16 (6), 34. 10.1007/s11934-015-0509-7 25894495

[B208] Song E. Zhu P. Lee S.-K. Chowdhury D. Kussman S. Dykxhoorn D. M. (2005). Antibody mediated *in vivo* delivery of small interfering RNAs via cell-surface receptors. Nat. Biotechnol. 23 (6), 709–717. 10.1038/nbt1101 15908939

[B209] Soutschek J. Akinc A. Bramlage B. Charisse K. Constien R. Donoghue M. (2004). Therapeutic silencing of an endogenous gene by systemic administration of modified siRNAs. Nature 432 (7014), 173–178. 10.1038/nature03121 15538359

[B210] Spisek R. Kukreja A. Chen L.-C. Matthews P. Mazumder A. Vesole D. (2007). Frequent and specific immunity to the embryonal stem cell–associated antigen SOX2 in patients with monoclonal gammopathy. J. Exp. Med. 204 (4), 831–840. 10.1084/jem.20062387 17389240 PMC2118551

[B211] Stenberg V. Y. Larsen R. H. Ma L.-W. Peng Q. Juzenas P. Bruland Ø. S. (2021). Evaluation of the PSMA-binding ligand 212Pb-NG001 in multicellular tumour spheroid and mouse models of prostate cancer. Int. J. Mol. Sci. 22 (9), 4815. 10.3390/ijms22094815 34062920 PMC8124365

[B212] Sugo T. Terada M. Oikawa T. Miyata K. Nishimura S. Kenjo E. (2016). Development of antibody-siRNA conjugate targeted to cardiac and skeletal muscles. J. Control. Release 237, 1–13. 10.1016/j.jconrel.2016.06.036 27369865

[B213] Sun Y. Davis E. (2021). Nanoplatforms for targeted stimuli-responsive drug delivery: a review of platform materials and stimuli-responsive release and targeting mechanisms. Nanomaterials 11 (3), 746. 10.3390/nano11030746 33809633 PMC8000772

[B214] Sundaram P. Abrahamse H. (2020). Effective Photodynamic Therapy for Colon Cancer Cells Using Chlorin e6 Coated Hyaluronic Acid-Based Carbon Nanotubes. Int. J. Mol. Sci. 21 (13), 4745. 10.3390/ijms21134745 32635295 PMC7369763

[B215] Syed Y. Y. (2021). Givosiran: a review in acute hepatic porphyria. Drugs 81 (7), 841–848. 10.1007/s40265-021-01511-3 33871817

[B216] Tan B. J. Y. Sow C. H. Koh T. S. Chin K. C. Wee A. T. S. Ong C. K. (2005). Fabrication of size-tunable gold nanoparticles array with nanosphere lithography, reactive ion etching, and thermal annealing. J. Phys. Chem. B 109 (22), 11100–11109. 10.1021/jp045172n 16852354

[B217] Tang C. Wang X. Soh H. Seyedin S. Cortez M. A. Krishnan S. (2014). Combining radiation and immunotherapy: a new systemic therapy for solid tumors? Cancer Immunol. Res. 2 (9), 831–838. 10.1158/2326-6066.cir-14-0069 25187273 PMC5367158

[B218] Tao Y. Chan H. F. Shi B. Li M. Leong K. W. (2020). Light: a magical tool for controlled drug delivery. Adv. Funct. Mater. 30 (49), 2005029. 10.1002/adfm.202005029 34483808 PMC8415493

[B219] Tenchov R. Bird R. Curtze A. E. Zhou Q. (2021). Lipid Nanoparticles─From liposomes to mRNA vaccine delivery, a landscape of research diversity and advancement. ACS Nano 15 (11), 16982–17015. 10.1021/acsnano.1c04996 34181394

[B220] Tewabe A. Abate A. Tamrie M. Seyfu A. Abdela Siraj E. (2021). Targeted drug delivery - from magic bullet to nanomedicine: principles, challenges, and future perspectives. J. Multidiscip. Healthc. 14, 1711–1724. 10.2147/jmdh.s313968 34267523 PMC8275483

[B221] Thangam R. Patel K. D. Kang H. Paulmurugan R. (2021). Advances in engineered polymer nanoparticle tracking platforms towards cancer immunotherapy-current status and future perspectives. Vaccines (Basel) 9 (8), 935. 10.3390/vaccines9080935 34452059 PMC8402739

[B222] Thangam R. Paulmurugan R. Kang H. (2022). Functionalized nanomaterials as tailored theranostic agents in brain imaging. Nanomaterials 12 (1), 18. 10.3390/nano12010018 PMC8746658 35009968

[B223] Tornesello A. L. Tagliamonte M. Buonaguro F. M. Tornesello M. L. Buonaguro L. (2022). Virus-like particles as preventive and therapeutic cancer vaccines. Vaccines (Basel) 10 (2), 227. 10.3390/vaccines10020227 35214685 PMC8879290

[B224] Ullah M. Wahab A. Khan S. U. Naeem M. ur Rehman K. Ali H. (2023). 3D printing technology: a new approach for the fabrication of personalized and customized pharmaceuticals. Eur. Polym. J. 195, 112240. 10.1016/j.eurpolymj.2023.112240

[B225] Umbricht C. A. Benešová M. Schibli R. Müller C. (2018). Preclinical development of novel PSMA-targeting radioligands: modulation of albumin-binding properties to improve prostate cancer therapy. Mol. Pharm. 15 (6), 2297–2306. 10.1021/acs.molpharmaceut.8b00152 29684274

[B226] Umbricht C. A. Köster U. Bernhardt P. Gracheva N. Johnston K. Schibli R. (2019). Alpha-PET for prostate cancer: preclinical investigation using 149Tb-PSMA-617. Sci. Rep. 9 (1), 17800. 10.1038/s41598-019-54150-w 31780798 PMC6882876

[B227] Vaughn B. A. Koller A. J. Chen Z. Ahn S. H. Loveless C. S. Cingoranelli S. J. (2021). Homologous structural, chemical, and biological behavior of Sc and Lu complexes of the picaga bifunctional chelator: toward development of matched theranostic pairs for radiopharmaceutical applications. Bioconjugate Chem. 32 (7), 1232–1241. 10.1021/acs.bioconjchem.0c00574 33284001

[B228] Wan J. Ren L. Li X. He S. Fu Y. Xu P. (2023). Photoactivatable nanoagonists chemically programmed for pharmacokinetic tuning and *in situ* cancer vaccination. Proc. Natl. Acad. Sci. 120 (8), e2210385120. 10.1073/pnas.2210385120 36787350 PMC9974508

[B229] Wang B. Pei J. Xu S. Liu J. Yu J. (2023). Recent advances in mRNA cancer vaccines: meeting challenges and embracing opportunities. Front. Immunol. 14, 1246682. 10.3389/fimmu.2023.1246682 37744371 PMC10511650

[B230] Wang J. Li B. Qiu L. Qiao X. Yang H. (2022). Dendrimer-based drug delivery systems: history, challenges, and latest developments. J. Biol. Eng. 16 (1), 18. 10.1186/s13036-022-00298-5 35879774 PMC9317453

[B231] Wang L. Shi J. Liu R. Liu Y. Zhang J. Yu X. (2014). Photodynamic effect of functionalized single-walled carbon nanotubes: a potential sensitizer for photodynamic therapy. Nanoscale 6 (9), 4642–4651. 10.1039/c3nr06835h 24647856

[B232] Wang P. Yin T. Li J. Zheng B. Wang X. Wang Y. (2016). Ultrasound-responsive microbubbles for sonography-guided siRNA delivery. Nanomedicine Nanotechnol. Biol. Med. 12 (4), 1139–1149. 10.1016/j.nano.2015.12.361 26733262

[B233] Wang T.-Y. Choe J. W. Pu K. Devulapally R. Bachawal S. Machtaler S. (2015). Ultrasound-guided delivery of microRNA loaded nanoparticles into cancer. J. Control. Release 203, 99–108. 10.1016/j.jconrel.2015.02.018 25687306 PMC4373966

[B234] Wang W. Jin Y. Liu X. Chen F. Zheng X. Liu T. (2021). Endogenous stimuli-activatable nanomedicine for immune theranostics for cancer. Adv. Funct. Mater. 31 (26), 2100386. 10.1002/adfm.202100386

[B235] Wang Y. Sun L. Mei Z. Zhang F. He M. Fletcher C. (2020). 3D printed biodegradable implants as an individualized drug delivery system for local chemotherapy of osteosarcoma. Mater. and Des. 186, 108336. 10.1016/j.matdes.2019.108336

[B236] Webb M. S. Tortora N. Cremese M. Kozlowska H. Blaquiere M. Devine D. V. (2001). Toxicity and toxicokinetics of a phosphorothioate oligonucleotide against the c-myc oncogene in cynomolgus monkeys. Antisense Nucleic Acid. Drug Dev. 11 (3), 155–163. 10.1089/108729001300338681 11446591

[B237] Wells C. M. Harris M. Choi L. Murali V. P. Guerra F. D. Jennings J. A. (2019). Stimuli-responsive drug release from smart polymers. J. Funct. Biomaterials 10 (3), 34. 10.3390/jfb10030034 PMC6787590 31370252

[B238] Wen R. Umeano A. C. Kou Y. Xu J. Farooqi A. A. (2019). Nanoparticle systems for cancer vaccine. Nanomedicine 14 (5), 627–648. 10.2217/nnm-2018-0147 30806568 PMC6439506

[B239] Wibowo D. Jorritsma S. H. T. Gonzaga Z. J. Evert B. Chen S. Rehm B. H. A. (2021). Polymeric nanoparticle vaccines to combat emerging and pandemic threats. Biomaterials 268, 120597. 10.1016/j.biomaterials.2020.120597 33360074 PMC7834201

[B240] Wilson A. N. Guiseppi-Elie A. (2013). Bioresponsive hydrogels. Adv. Healthc. Mater. 2 (4), 520–532. 10.1002/adhm.201200332 23233355

[B241] Wilson B. Geetha K. M. (2022). Lipid nanoparticles in the development of mRNA vaccines for COVID-19. J. Drug Deliv. Sci. Technol. 74, 103553. 10.1016/j.jddst.2022.103553 35783677 PMC9238147

[B242] Wolfrum C. Shi S. Jayaprakash K. N. Jayaraman M. Wang G. Pandey R. K. (2007). Mechanisms and optimization of *in vivo* delivery of lipophilic siRNAs. Nat. Biotechnol. 25 (10), 1149–1157. 10.1038/nbt1339 17873866

[B243] Wong E. Goldberg T. (2014). Mipomersen (kynamro): a novel antisense oligonucleotide inhibitor for the management of homozygous familial hypercholesterolemia. P t 39 (2), 119–122. 24669178 PMC3956393

[B244] Wranik M. Weinert T. Slavov C. Masini T. Furrer A. Gaillard N. (2023). Watching the release of a photopharmacological drug from tubulin using time-resolved serial crystallography. Nat. Commun. 14 (1), 903. 10.1038/s41467-023-36481-5 36807348 PMC9936131

[B245] Wu M. Huang S. (2017). Magnetic nanoparticles in cancer diagnosis, drug delivery and treatment. Mol. Clin. Oncol. 7 (5), 738–746. 10.3892/mco.2017.1399 29075487 PMC5649002

[B246] Xueling Ren J. L. Wang X. Liu X. Meng E. Zhang R. Sang Y. (2017). Photoactivatable RNAi for cancer gene therapy triggered by near-infrared-irradiated single-walled carbon nanotubes. Int. J. Nanomedicine 12, 7885–7896. 10.2147/IJN.S141882 29138556 PMC5666115

[B247] Yang H. Deng L. Li T. Shen X. Yan J. Zuo L. (2015). Multifunctional PLGA nanobubbles as theranostic agents: combining doxorubicin and P-gp siRNA Co-delivery into human breast cancer cells and ultrasound cellular imaging. J. Biomed. Nanotechnol. 11 (12), 2124–2136. 10.1166/jbn.2015.2168 26510307

[B248] Yang Q. Jacobs T. M. McCallen J. D. Moore D. T. Huckaby J. T. Edelstein J. N. (2016). Analysis of pre-existing IgG and IgM antibodies against polyethylene glycol (PEG) in the general population. Anal. Chem. 88 (23), 11804–11812. 10.1021/acs.analchem.6b03437 27804292 PMC6512330

[B249] Yang Y. Qiao X. Huang R. Chen H. Shi X. Wang J. (2020). E-jet 3D printed drug delivery implants to inhibit growth and metastasis of orthotopic breast cancer. Biomaterials 230, 119618. 10.1016/j.biomaterials.2019.119618 31757530

[B250] Yi J. S. Cox M. A. Zajac A. J. (2010). T-cell exhaustion: characteristics, causes and conversion. Immunology 129 (4), 474–481. 10.1111/j.1365-2567.2010.03255.x 20201977 PMC2842494

[B251] Yin M. Tong J. Meng F. Liu C. Liu X. Fang F. (2022). Near-infrared-II activatable symbiotic 2D carbon–clay nanohybrids for dual imaging-guided combinational cancer therapy. ACS Appl. Mater. and Interfaces 14 (44), 49471–49482. 10.1021/acsami.2c11340 36301911

[B252] Zhang D. Tian S. Liu Y. Zheng M. Yang X. Zou Y. (2022a). Near infrared-activatable biomimetic nanogels enabling deep tumor drug penetration inhibit orthotopic glioblastoma. Nat. Commun. 13 (1), 6835. 10.1038/s41467-022-34462-8 36369424 PMC9652403

[B253] Zhang H. Yang J. Sun R. Han S. Yang Z. Teng L. (2023b). Microfluidics for nano-drug delivery systems: from fundamentals to industrialization. Acta Pharm. Sin. B 13 (8), 3277–3299. 10.1016/j.apsb.2023.01.018 37655333 PMC10466004

[B254] Zhang Y. Chen J. Shi L. Ma F. (2023a). Polymeric nanoparticle-based nanovaccines for cancer immunotherapy. Mater. Horizons 10 (2), 361–392. 10.1039/d2mh01358d 36541078

[B255] Zhang Y. Tian S. Huang L. Li Y. Lu Y. Li H. (2022b). Reactive oxygen species-responsive and Raman-traceable hydrogel combining photodynamic and immune therapy for postsurgical cancer treatment. Nat. Commun. 13 (1), 4553. 10.1038/s41467-022-32160-z 35931666 PMC9356008

[B256] Zhao L. Meng F. Li Y. Liu S. Xu M. Chu F. (2023). Multivalent nanobody conjugate with rigid, reactive oxygen species scavenging scaffold for multi-target therapy of alzheimer's disease. Adv. Mater. 35 (17), 2210879. 10.1002/adma.202210879 36786375

[B257] Zhao S. Yu X. Qian Y. Chen W. Shen J. (2020). Multifunctional magnetic iron oxide nanoparticles: an advanced platform for cancer theranostics. Theranostics 10 (14), 6278–6309. 10.7150/thno.42564 32483453 PMC7255022

[B258] Zhao X. Yuan C. Wangmo D. Subramanian S. (2021b). Tumor-secreted extracellular vesicles regulate T-cell costimulation and can Be manipulated to induce tumor-specific T-cell responses. Gastroenterology 161 (2), 560–574.e11. 10.1053/j.gastro.2021.04.036 33895168

[B259] Zhao Y. Baldin A. V. Isayev O. Werner J. Zamyatnin A. A. Bazhin A. V. (2021a). Cancer vaccines: antigen selection strategy. Vaccines 9 (2), 85. 10.3390/vaccines9020085 33503926 PMC7911511

[B260] Zheng M. Liu Y. Wang Y. Zhang D. Zou Y. Ruan W. (2019). ROS-responsive polymeric siRNA nanomedicine stabilized by triple interactions for the robust glioblastoma combinational RNAi therapy. Adv. Mater. 31 (37), 1903277. 10.1002/adma.201903277 31348581

[B261] Zhou X. Zhao B. Wang L. Yang L. Chen H. Chen W. (2023). A glucose-responsive nitric oxide release hydrogel for infected diabetic wounds treatment. J. Control. Release 359, 147–160. 10.1016/j.jconrel.2023.05.047 37277053

[B262] Zhu X.-J. Li R.-F. Xu L. Yin H. Chen L. Yuan Y. (2019). A novel self-assembled mitochondria-targeting protein nanoparticle acting as theranostic platform for cancer. Small 15 (2), 1803428. 10.1002/smll.201803428 30450734

[B263] Zhu Y. Zhu L. Wang X. Jin H. (2022). RNA-based therapeutics: an overview and prospectus. Cell Death and Dis. 13 (7), 644. 10.1038/s41419-022-05075-2 PMC9308039 35871216

[B264] Zhu Y.-D. Chen S.-P. Zhao H. Yang Y. Chen X.-Q. Sun J. (2016). PPy@MIL-100 nanoparticles as a pH- and near-IR-irradiation-responsive drug carrier for simultaneous photothermal therapy and chemotherapy of cancer cells. ACS Appl. Mater. and Interfaces 8 (50), 34209–34217. 10.1021/acsami.6b11378 27998104

